# Targeting the SYVN1-EGFR axis: a breakthrough strategy for TKI-resistant NSCLC

**DOI:** 10.1038/s41419-025-07978-2

**Published:** 2025-08-28

**Authors:** Xinsheng Xie, Weilai Tong, Yue Xie, Haoxin Jiang, Alan Jiang, Junming Huang, Zhili Liu, Jingjing Yu

**Affiliations:** 1 Department of Orthopedic Surgery, The Third Affiliated Hospital, Jiangxi Medical College, Nanchang University, Nanchang, China; 2 School of Information Management, Jiangxi University of Finance and Economics, Nanchang, China; 3 Jiangxi Provincial Key Laboratory of Spine and Spinal Cord Diseases, Nanchang, China; 4 Department of Orthopedic Surgery, The First Affiliated Hospital, Jiangxi Medical College, Nanchang University, Nanchang, China; 5 Department of Urology, The First Affiliated Hospital, Jiangxi Medical College, Nanchang University, Nanchang, China; 6 Department of Respiratory and Critical Care Medicine, Jiangxi Provincial Key Laboratory of Respiratory Diseases, Jiangxi Institute of Respiratory Diseases, Jiangxi Clinical Research Center for Respiratory Diseases, The First Affiliated Hospital, Jiangxi Medical College, Nanchang University, Nanchang, China

**Keywords:** Non-small-cell lung cancer, Oncogenes

## Abstract

Non-small cell lung cancer (NSCLC) is the leading cause of cancer-related death. Currently, molecular targeted therapy remains a crucial approach to the treatment of NSCLC. However, the development of acquired drug resistance poses significant challenges for subsequent treatment. Identifying new therapeutic targets is of great significance for improving the prognosis of patients with NSCLC. Here, we verify synoviolin-1 (SYVN1) as a potential new therapeutic target for NSCLC. SYVN1 is highly expressed in NSCLC, and its upregulation is associated with poor prognosis. We show that the N-terminus (1–290 aa) of SYVN1 directly interacts with the intracellular domain of the epidermal growth factor receptor (EGFR) and activates EGFR signaling, promoting NSCLC growth in vitro and in vivo. Specifically, SYVN1 facilitates Lys 63-linked ubiquitination of EGFR and inhibits proteasome-mediated EGFR degradation. Moreover, we found that SYVN1 inhibits EGFR endocytosis, thereby increasing the amount of EGFR on the cell membrane. Furthermore, we confirmed that LS-102, an enzyme activity inhibitor of SYVN1, inhibits cell proliferation induced by SYVN1. Significantly, LS-102 in combination with the EGFR-TKI AZD9291 exhibits strong inhibitory effects on NSCLC growth and reverses the resistance of NSCLC to AZD9291. Together, our study demonstrates that the SYVN1-EGFR axis plays a critical role in NSCLC development and suggests that targeting the SYVN1-EGFR axis to destabilize EGFR may represent a putative therapeutic strategy for TKI-resistant NSCLC.

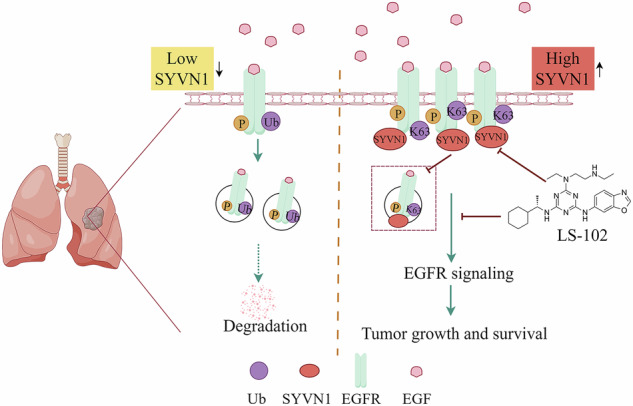

## Introduction

Non-small cell lung cancer (NSCLC) is the most prevalent subtype of lung cancer and is recognized as the leading cause of cancer-related mortality worldwide [1, 2]. Despite advancements in early detection and targeted therapies, the emergence of various gene mutations has led to a poor prognosis for NSCLC. Activation of epidermal growth factor receptor (EGFR) signaling, driven by EGFR gene amplification and point mutations such as L858R and T790M, is the most common driving factor in NSCLC, accounting for ~90% of lung cancer cases [3, 4]. Molecular therapy targeting EGFR with tyrosine kinase inhibitors (TKIs) has revolutionized the treatment landscape for NSCLC [5, 6]. Gefitinib and afatinib, the first- and second-generation EGFR-TKIs, are used as first-line therapy for advanced NSCLC patients harboring EGFR sensitizing mutations [7] (exon 19 deletion and L858R mutation). However, the emergence of new mutations leads to resistance against existing TKIs in patients, thereby limiting their long-term efficacy and presenting significant challenges for clinicians [8]. The classical T790M mutation, which introduces steric hindrance at the ATP-binding pocket, reduces the binding affinity of TKIs, thereby conferring resistance to first- and some second-generation TKIs [9]. Osimertinib (AZD9291), a third-generation EGFR-TKI, effectively overcomes the common T790M drug resistance mutation. Subsequent studies identified the C797S mutation as a key resistance mechanism against osimertinib, as it alters the covalent binding site and prevents effective drug engagement [10]. So far, there are no effective treatment options available for patients with EGFR^L718Q^ and EGFR^C797S^ mutations [11, 12]. Current research efforts are primarily directed toward overcoming EGFR mutation-associated resistance through the development of novel agents and combination strategies [13]. These efforts leverage insights from pharmacodynamics and structural biology to design more effective inhibitors. Additionally, researchers are exploring combination therapies that incorporate immunotherapy to extend progression-free survival. These advancements provide promising avenues for precision medicine approaches to counteract EGFR mutation-induced resistance in NSCLC. Therefore, elucidating novel mechanisms that regulate EGFR and identifying new therapeutic targets are essential for improving patient outcomes and overcoming resistance mechanisms in NSCLC.

EGFR is a member of the ErbB family of receptor tyrosine kinases, which are involved in regulating cell proliferation, differentiation, survival, and migration [14]. In physiological conditions, the binding of the epidermal growth factor (EGF) ligand induces receptor dimerization and autophosphorylation, after which the receptor is translocated from the cell membrane to the cytoplasm. In the cytoplasm, the phosphorylated EGFR recruits GRB2, which, in turn, recruits SOS to RAS, activating the downstream RAS/RAF/MEK/ERK and PI3K/AKT signaling pathways [15]. The fate of the EGFR protein and the activity of EGFR signaling are intricately and precisely regulated by positive and negative feedback loops. On the one hand, GRB2 recruits the ubiquitin E3 ligase c-Cbl to ubiquitinate EGFR and transport it to the endosome. The ubiquitinated EGFR then recruits HRS and is subsequently transported to the lysosome for degradation, resulting in the attenuation of signaling. Additionally, protein phosphatases can dephosphorylate EGFR, which also leads to the attenuation of signaling. On the other hand, deubiquitinating enzymes remove ubiquitin from ubiquitinated EGFR, thereby blocking its transport to the lysosome and allowing it to be recycled back to the cell membrane [16, 17]. The activity of the EGFR signaling pathway is tightly regulated by the balance of these processes. In certain pathological conditions, such as cancer, this balance is disrupted, resulting in increased stability of the EGFR protein and enhanced localization to the cell membrane [3]. Under certain circumstances, the regulatory mechanisms of EGFR become more complicated and dysfunctional, resulting in heightened signaling activity and swift disease progression. Therefore, elucidating the molecular mechanisms that regulate EGFR in these processes is expected to provide a new approach for treating diseases, including NSCLC.

Synoviolin-1 (SYVN1) is a member of the RING family of ubiquitinating enzymes that plays an important role in the endoplasmic reticulum-associated degradation (ERAD) pathway [18, 19]. It has been established that SYVN1 plays a crucial role in embryonic development, as well as in the processes of inflammation and immunity [20–22]. Recent studies have found that SYVN1 plays distinct roles in various cancers. In cancers of the breast and ovary, SYVN1 inhibits tumor progression by promoting the ubiquitination of substrate proteins, such as PFAK [23], CPT2 [24], and SLC7A11 [25], which can lead to either their degradation or stabilization. In liver cancer, SYVN1 promotes the proliferation, metastasis, and immune evasion of liver cancer cells through the ubiquitination and degradation of substrate proteins such as PTEN [26] and FoxO1 [27]. Similarly, SYVN1 also plays different roles through a variety of regulatory mechanisms in the cervical [28], colon [29], pancreas [30], thyroid [31] and other tumors [32]. In lung cancer, SYVN1 ubiquitylation degrades substrates such as SIRT2 [33] and ATG3 [34], and it also regulates the membrane localization of the MCT4 [35] protein to promote tumor progression. Taken together, the oncogenic role of SYVN1 makes it a promising therapeutic target, and efforts are underway to develop selective inhibitors, particularly LS-102, to disrupt its function [36]. In this study, we demonstrate that SYVN1 functions as an E3 ubiquitin ligase for EGFR, facilitating the formation of Lys 63-linked polyubiquitin chains on the EGFR protein. This process blocks proteasome-mediated degradation, thereby promoting EGFR-driven NSCLC growth. Additionally, SYVN1 increases the amount of EGFR on the cell membrane by inhibiting its endocytosis, resulting in continuous activation of EGFR signaling. Moreover, the SYVN1 enzyme activity inhibitor LS-102, which has potential applications in the study of rheumatoid arthritis and obesity [37–39], inhibits cell proliferation induced by SYVN1. Significantly, the combination of LS-102 with AZD9291 shows potent inhibitory effects on NSCLC and reverses resistance to AZD9291. Targeting the SYVN1-EGFR axis to destabilize EGFR could potentially offer a promising therapeutic strategy for treating TKI-resistant NSCLC.

## Materials and methods

### Cell culture, cells transfection, and reagents

The HEK293T cells and human NSCLC cell lines A549, H1299, PC9, and H1975 were purchased from the American Type Culture Collection (ATCC, Manassas, VA, USA). All cell lines were authenticated by STR profiling and tested to be mycoplasma-free. HEK293T and H1299 cells were grown in DMEM medium (Gibco, NY, USA) supplemented with 10% FBS and penicillin (100 U/mL)/streptomycin (100 μg/mL). The A549, H1975, and PC9 cells were grown in RPMI-1640 medium (Gibco, NY, USA) supplemented with 10% FBS and penicillin (100 U/mL)/streptomycin (100 μg/mL). Cells were cultured in a 37 °C incubator with 5% CO_2_.

For HEK293T transfection, cells were seeded in 6-well plates at appropriate densities to achieve a confluence of 50% to 60% at the time of transfection; Cells were transfected with various plasmids using polyethylenimine (PEI) (40820ES, YEASEN, Shanghai, China) at a mass-to-volume ratio of 1:2 and refreshed the medium 12 hours later. For H1299 transfection, cells were seeded in 6-well plates at appropriate densities to achieve a confluence of 70% to 90% at the time of transfection; Cells were transfected with plasmids or siRNA using Lipofectamine 3000 (Thermo, Waltham, MA, USA) (mass-to-volume ratio of 1:2) according to the manufacturer’s instructions and refreshed the medium 6 hours after transfection.

MG132 (HY-13259), cycloheximide (HY-12320), chloroquine (HY-17589A), LS-102 (HY-135844), protease inhibitor cocktail (HY-K0010), and phosphatase inhibitor cocktail (HY-K0022) were purchased from MedChemExpress (Shanghai, China). AZD9291 (S7297) was obtained from Selleck Chemicals (HOU, USA). Recombinant Human EGF (236-EG) was purchased from R&D Systems (MN, USA).

### Plasmids and lentiviral infection

Ubiquitin (Ub) expression vectors with an HA tag, SYVN1 plasmids carrying HA (P35682), Flag (P42971), or His (P43837) tags, as well as EGFR eukaryotic expression plasmids with a Flag tag and lentiviral plasmids containing T790M/C797S mutations, were obtained from MiaoLingBio (Wuhan, China). All truncated and point mutation plasmids of SYVN1 and EGFR were constructed using the corresponding wild-type plasmids as templates, following the guidelines of the KOD-Plus Mutagenesis kit (SMK-101, Toyobo, Osaka).

The LentiX construct (pGV492-GFP) for SYVN1 (NM_172230) overexpression and the Lenti X-shRNA construct (pGV298-Cherry) for shRNA-SYVN1 knockdown were generated, packaged, and purified by GeneChem. The shRNA target sequences were as follows: shCon, 5’-TTCTCCGAACGTGTCACGT-3’; shSYVN1#1, 5’-AATGCTT-AATCCCGGGAAA-3’; shSYVN1#2, 5’-GCTGTGACAGATGCCATCA-3’. The LentiX construct (pLV3) for PERK (NM_004836) overexpression was generated, packaged, and purified by MiaoLingBio (Wuhan, China). Lentivirus infection was performed according to the protocol provided by the manufacturer.

### Western blot analysis

Total proteins from the cells were extracted by lubrol lysis buffer (0.5% lubrol-PX, 50 mM KCl, 2 mM CaCl_2_, 20% glycerol, 50 mM Tris-HCl, pH 7.4), supplemented with a phosphatase/protease inhibitor cocktail. Equal amounts of proteins were loaded onto a 10% SDS-PAGE gel for separation, with the upper layer running at 50 V and the lower layer running at 100 V. The separated proteins were transferred from the gel to a 0.22 μm PVDF membrane using a sandwich clamp at a current of 200 mA in ice-cold transfer solution at the end of the gel run for 2 hours. The membranes loaded with proteins were blocked with 5% non-fat milk at room temperature for 30 minutes, followed by overnight incubation with the primary antibody at 4°C. The next day, the membranes were washed with TBST (0.5% Tween-20) three times for 10 minutes each, and then the membranes were incubated with the secondary antibody at room temperature for 1 hour. The membranes were washed, and the blots were imaged using the Bio-Rad ChemiDoc MP system.

Antibodies specific for either EGFR (4267, 1:1000), p-EGFR (1068) (3777, 1:1000), ERK1/2 (4695, 1:1000), p-ERK1/2 (4370, 1:1000), AKT (9272, 1:1000), or p-AKT (4060, 1:1000) were purchased from Cell Signaling Technology (CST, MA, USA). Antibodies specific for either SYVN1 (13473-1-AP, 1:1000), HA (51064-2-AP, 1:1000), Flag (20543-1-AP, 1:1000), His (66005-1-Ig, 1:1000), Ubiquitin (10201-2-AP, 1:1000), Bcl-XL (10783-1-AP, 1:2000), BAX (50599-2-Ig, 1:2000), CHOP (15204-1-AP, 1:1000), ATF4 (10835-1-AP, 1:1000), p-PERK (29546-1-AP, 1:1000), PERK (20582-1-AP, 1:1000), XBP1S (24168-1-AP, 1:1000), IRE1α (27528-1-AP, 1:1000), ATF6 (24169-1-AP, 1:2000), p-EIF2S1 (68023-1-Ig, 1:5000), EIF2S1 (11170-1-AP, 1:5000), or GAPDH (60004-1-Ig, 1:1000) were purchased from Proteintech (Wuhan, China). Protein A/G magnetic beads (HY-K0202) and HA magnetic beads (HY-K0201) were purchased from MedChemExpress (Shanghai, China). Flag M2 magnetic beads (M8823) were purchased from Sigma (MO, USA).

### Cell proliferation assay

CCK-8 and colony formation assays were conducted to evaluate cell proliferation and viability. For the CCK-8 assay, 1 × 10^3^ cells/well were seeded in 96-well plates. Cell viability was assessed by measuring the OD450 using the Cell Counting Kit-8 (40203ES76, YESEN, Shanghai, China). Finally, the cell viability curve was established using GraphPad Prism software (Prism 8).

For the colony formation assay, 1 × 10^3^ cells were plated in six-well plates per well and cultured for 10 days. The colonies were fixed with 4% paraformaldehyde for 10 minutes and stained with 0.1% crystal violet for 10 minutes at room temperature. Colonies containing more than 50 cells were counted using ImageJ.

### Immunoprecipitation

To verify the interaction between SYVN1 and EGFR, HA-SYVN1 and Flag-EGFR plasmids were co-transfected into HEK293T cells by using PEI. After 48 hours of transfection, cells were collected and lysed by cold lubrol lysis buffer on ice to obtain the protein supernatants. One-tenth of the supernatants was set aside as a loading control for subsequent analyses, and the remaining supernatants were transferred to EP tubes containing Flag or HA magnetic beads that had been pre-washed with PBST (0.1% Tween-20) and incubated for 2 hours at 4 °C. After incubation, the supernatants were discarded after centrifugation at 1000 rpm, and the Flag or HA magnetic beads were washed six times by adding PBST, gently inverting and mixing. An appropriate amount of loading buffer was added to the magnetic beads, and the samples were boiled in a metal bath at 95 °C for 10 minutes before Western blot analysis.

For endogenous immunoprecipitation, H1299 and H1975 cells were cultured and lysed using lubrol lysis buffer to obtain protein supernatant. One-tenth of the supernatants was reserved as a loading control for subsequent analyses, and the remaining supernatants were incubated with 1 µg of SYVN1 (13473-1-AP, Proteintech, China) primary antibody or isotype-matched IgG at 4 °C overnight. The next day, the supernatants containing the antibody-bound complexes were transferred to EP tubes containing protein A/G magnetic beads pre-washed with PBST (0.1% Tween-20) and incubated at 4 °C for 2 hours. After incubation, the supernatants were discarded after centrifugation at 1000 rpm, and the protein A/G magnetic beads were washed six times by adding PBST, gently inverting and mixing. An appropriate amount of loading buffer was added to the magnetic beads, and the samples were boiled in a metal bath at 95 °C for 10 minutes before Western blot analysis.

### MBP-pulldown assay

To assess the physical interaction between SYVN1 and EGFR, MBP, MBP-EGFR (695-1022 aa), and Flag-SYVN1 were purified separately. The MBP or MBP-EGFR (695-1022 aa)-coated amylose resin was then incubated with Flag-SYVN1, and the precipitated proteins were analyzed by Western blot.

### Protein half-life analysis

H1299 cells overexpressing SYVN1 and control cells were seeded in six well plates. Once the cells reached 80% confluence, they were switched to fresh serum-containing or serum-free medium, and cycloheximide was added at a concentration of 50 μg/mL at various time points. EGFR protein abundance was assessed by Western blot.

### Ubiquitination assay

In H1299 cells with SYVN1 overexpression or knockdown, and in HEK293T cells co-transfected with plasmids expressing His-SYVN1, Flag-EGFR, and either wild-type HA-Ub, HA-Ub-K48, HA-Ub-K63, HA-Ub-K48R, or HA-Ub-K63R, the cells were treated with 20 μM MG132 for 6 hours before harvesting. After cell lysis, EGFR protein was enriched by immunoprecipitation, and the level of EGFR ubiquitination was detected by Western blot.

For the in vitro ubiquitination assay, all procedures were conducted according to the manufacturer’s instructions. Briefly, the amylose resin coated with MBP-EGFR (695–1022 aa) was incubated with purified Flag-SYVN1 or Flag for 2 hours at room temperature, along with the addition of E1 and E2 enzymes, ATP, and ubiquitin at the recommended concentrations. Excess components were washed away, leaving the resin, and the ubiquitination levels of the precipitated EGFR protein were analyzed by Western blot.

### EGFR biotinylation assay

Biotin labeling of membrane proteins was performed following previously reported protocols [40]. Briefly, H1299 cells were serum-starved overnight and then stimulated with 20 ng/mL EGF for varying durations. The cells were washed with ice-cold PBS (pH 8.0) and incubated on ice with 0.5 mg/mL sulfo-NHS-SS-Biotin (21331, Thermo Scientific) in PBS (pH 8.0) for 30 minutes. The cells were then washed twice with ice-cold PBS (pH 8.0), and excess sulfo-NHS-SS-biotin was blocked with 50 mM NH_4_Cl. Finally, after cell lysis, biotinylated proteins were captured from equal amounts of cell extracts using Streptavidin-coated Dynabeads (Invitrogen) at 4 °C, for 4 hours, and analyzed by Western blot.

### Immunofluorescence staining

To determine the spatial distribution of EGFR and SYVN1, H1299 cells were seeded at ~20% confluence into 24-well plates precovered with glass coverslips and cultured overnight. The cells were fixed with 4% paraformaldehyde for 10 minutes at room temperature, followed by permeabilization with 0.5% Triton X-100 at room temperature for an additional 10 minutes. Subsequently, the cells were blocked with 10% BSA at room temperature for 1 hour. Primary antibodies against SYVN1 (13473-1-AP, Proteintech, Rabbit, 1:200) and EGFR (66455-1-Ig, Proteintech, Mouse, 1:100), diluted in 2% BSA, were added to the well and incubated overnight at 4°C. After incubation with the primary antibodies, the cells were washed three times with PBST for 10 minutes each, followed by a 1-hour incubation at room temperature with the AF488 (A0423, Beyotime, Rabbit, 1:500) and AF555 (A0460, Beyotime, Mouse, 1:500) secondary antibodies. The nuclei were stained with DAPI (C1002, Beyotime, 1:500). The cells were observed under a laser confocal microscope for imaging (Stellaris 5, Leica).

To determine the spatial distribution of EGFR and EEA1 or M6PR, H1299 cells were seeded at approximately 50% confluence into 24-well plates precovered with glass coverslips and cultured until adherent. Cells were then serum-starved overnight and stimulated with 20 ng/mL EGF for varying durations. The next steps are the same as above. Primary antibodies EGFR (4267, CST, Rabbit, 1:200), EEA1 (ab70521, Abcam, Mouse, 1:500), and M6PR (ab2733, Abcam, Mouse, 1:500), as well as secondary antibodies AF555 (A0460, Beyotime, Mouse, 1:500) and AF647 (A0468, Beyotime, Rabbit, 1:500), were used in staining.

### Animal experiments

H1299 (Lv-Con) and H1299/AZDR (Lv-AZDR) (1 × 10^7^) cells were injected subcutaneously into the left and right groin of 5-week-old female BALB/c nude mice (*n* = 40), respectively. Forty nude mice were randomly divided into four treatment groups: DMSO, AZD9291, LS-102, AZD9291/LS-102. Mice were administered AZD9291 (5 mg/kg, via daily gastric gavage in PBS containing 5% DMSO, 40% PEG 300, and 5% Tween-80) or LS-102 (3 mg/kg, via daily intraperitoneal injection in PBS containing 10% DMSO, 40% PEG 300, and 5% Tween-80) twice a week for 3 weeks. The weight and health of the mice were closely monitored every day, and the experiment was stopped as soon as the mice lost more than 20% of their weight or showed signs of poor health. Tumor sizes were measured using calipers every three days. At the experimental endpoint, the mice were euthanized by cervical dislocation, and the tumors were quickly excised, weighed and placed on ice. Tumor weights and volumes (calculated using the formula: (length × width^2^)/2) were presented as mean ± SD. Mice were housed in specific pathogen-free individually ventilated cages. All animal care and experiments were conducted in accordance with institutional ethical guidelines and were approved by the Animal Ethics Review Committee at the First Affiliated Hospital of Nanchang University.

### Immunohistochemistry staining

Human non-small cell lung cancer (NSCLC) tissue specimens were obtained from the lung cancer patients who had not received radiotherapy or chemotherapy and underwent surgical resection at the First Affiliated Hospital of Nanchang University. The study was approved by the Institutional Review Board of the First Affiliated Hospital of Nanchang University.

Immunohistochemistry (IHC) staining was performed according to the manufacturer’s instructions for the reagent kit (E-IR-R213, Elabscience). Briefly, antigen retrieval was conducted using Tris-EDTA (TE) buffer in a pressure cooker for 3 minutes. After cooling, the tissues were incubated with 3% hydrogen peroxide for 10 minutes to eliminate endogenous peroxidase activity and subsequently blocked with 10% normal goat serum at room temperature for 30 minutes. The tissues were then incubated overnight in a humidified chamber at 4 °C with anti-SYVN1 antibody (13473-1-AP, Proteintech, 1:30,000) or anti-EGFR antibody (GB111504, Servicebio, 1:2000), followed by visualization of the immunoreactivity. Images were obtained using a digital slide scanner (HS6, SOPTOP). Specimens were analyzed by three independent pathologists to obtain expression scores using the German semiquantitative scoring method. Each specimen was scored based on the staining intensity of the nuclei, cytoplasm, and membrane, using the following scale: no staining = 0, weak staining = 1, moderate staining = 2, and strong staining = 3. Additionally, the proportion of stained cells was assessed as follows: 0% = 0, 1–24% = 1, 25–49% = 2, 50–74% = 3, and 75–100% = 4. The final immunoreactive score was calculated as the product of the expression intensity score and the extent score.

Tissue microarray of human lung adenocarcinoma and paired adjacent normal tissues (HLugA180Su12) were purchased from Shanghai Outdo Biotech Company (Shanghai, China). The study was approved by the Ethics Committee of Shanghai Outdo Biotech Company.

### Statistical analysis

All experiments were conducted independently at least three times. The data were presented as mean ± SD. Differences between the two groups were assessed using independent samples *t* tests. All analyses were performed using SPSS v.13.0 (SPSS Inc., Chicago, IL) and GraphPad Prism 8.0 (GraphPad Software, San Diego, CA). Differences were considered statistically significant at *p* < 0.05. *p < 0.05; **p < 0.01; ***p < 0.001.

## Results

### SYVN1 promotes the proliferation of NSCLC cells

**Fig. 1:**
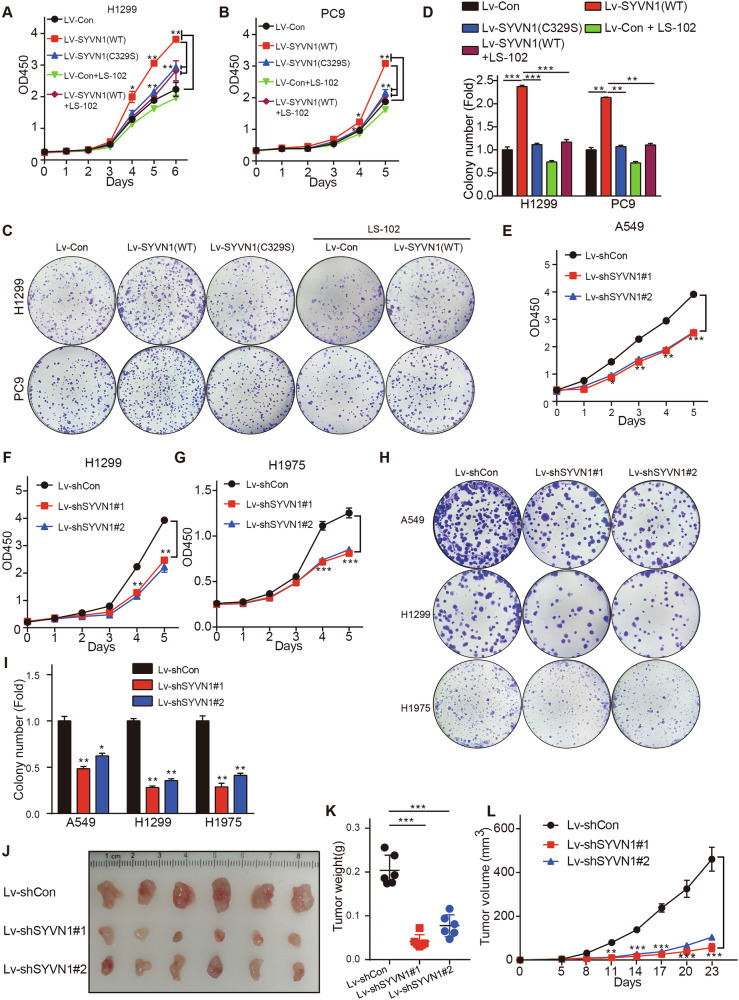
SYVN1 promotes the proliferation of NSCLC cells. **A**–**D** H1299 or PC9 cells stably expressing either WT or C329S mutant SYVN1, or treated with LS-102, were subjected to (**A**, **B**) CCK-8 assays or (**C**, **D**) colony formation assays. **E**–**I** A549, H1299, or H1975 cells stably expressing shSYVN1 were subjected to CCK-8 assays (**E**–**G**) or colony formation assays (**H**, **I**). **J**–**L** H1299 cells with SYVN1 knockdown were implanted subcutaneously into nude mice. At the experimental endpoint, the tumors were excised, photographed (**J**), and weighed (**K**). Tumor growth curves were then generated (**L**). **A**, **B**, **D**, **E**–**G**, **I**, **K**, **L** Data are presented as the mean ± SD. *P* values were determined by an independent samples *t-*test, *p < 0.05; **p < 0.01; ***p < 0.001.

To verify the exact role of SYVN1 in NSCLC progression, we overexpressed or knocked down SYVN1 in NSCLC cells and observed its effects on cell proliferation and tumor growth. The efficiency of SYVN1 overexpression and knockdown was validated through western blot (Fig. S1). As shown in fig. 1A, B, overexpression of wild-type (WT) SYVN1 promoted the proliferation of H1299 and PC9 cells. However, the C329S mutant SYVN1, which results in a loss of enzymatic activity, did not promote cell proliferation. Furthermore, concurrent treatment with the SYVN1 selective inhibitor LS-102 inhibited cell proliferation upon overexpression of WT SYVN1, whereas the controls were largely unaffected. Additionally, cell colony formation assays indicated that SYVN1 promoted cell proliferation, while the C329S mutant and LS-102 counteracted this effect (Fig. 1C, D). Conversely, CCK-8 and colony formation assays confirmed that the knockdown of SYVN1 inhibits the proliferation of NSCLC cells (Fig. 1E–I). In vivo, knockdown of SYVN1 significantly inhibited the growth of NSCLC tumors, as evidenced by smaller tumor sizes and a reduced growth rate (Fig. 1J–L). These data suggest that SYVN1 plays a role in promoting the proliferation of NSCLC cells, and that LS-102 is capable of inhibiting this effect.

### SYVN1 interacts with EGFR

**Fig. 2:**
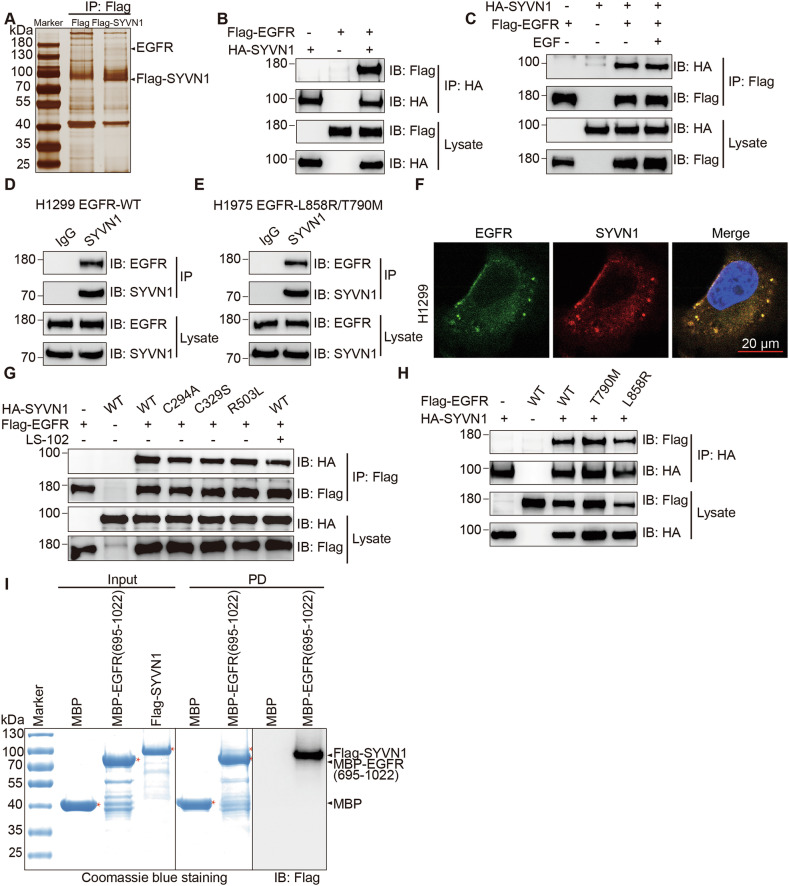
SYVN1 interacts with EGFR. **A** Flag-SYVN1 was enriched from H1299 cells overexpressing SYVN1 via immunoprecipitation, followed by mass spectrometry analysis to identify interacting proteins. EGFR may be one of the interacting proteins of SYVN1. **B**, **C** HA-SYVN1 and Flag-EGFR plasmids were co-transfected into HEK293T cells, and the interaction between SYVN1 and EGFR was validated by immunoprecipitation using either an HA antibody (**B**) or a Flag antibody (**C**). **D**, **E** Endogenous immunoprecipitation experiments were conducted in H1299 (**D**) and H1975 (**E**) cells to confirm the interaction between SYVN1 and EGFR. **F** SYVN1 co-localizes with EGFR in H1299 cells. **G** HA-SYVN1 (WT, C294A, C329S, or R503L) and Flag-EGFR plasmids were co-transfected into HEK293T cells, and the interaction between mutant SYVN1 and EGFR was validated by immunoprecipitation using a Flag antibody. **H** HA-SYVN1 and Flag-EGFR (WT, T790M, or L858R) plasmids were co-transfected into HEK293T cells, and the interaction between SYVN1 and mutant EGFR was validated by immunoprecipitation using an HA antibody. **I** MBP pull-down assays were performed to assess the direct interaction between SYVN1 and EGFR following their purification. All experiments were conducted independently three times.

To elucidate the potential mechanisms by which SYVN1 exerts its effects, we employed mass spectrometry to identify molecules that may interact with SYVN1. Among a series of proteins, we found that EGFR could be a potential interacting partner of SYVN1 (Fig. 2A). To verify their interaction, HEK293T cells were co-transfected with HA-SYVN1 and Flag-EGFR plasmids. Through exogenous immunoprecipitation experiments, we discovered an interaction between SYVN1 and EGFR, and found that EGF ligands do not affect this interaction (Fig. 2B, C). Subsequently, the endogenous interaction between SYVN1 and EGFR was confirmed in both H1299 and H1975 cells (Fig. 2D, E). Furthermore, using immunofluorescence staining, we observed a co-localization of SYVN1 and EGFR within H1299 cells (Fig. 2F). Additionally, we tested the interaction between EGFR and common mutants of SYVN1 (C294A, C329S, R503L) and found that the binding of SYVN1 mutants to EGFR was weakened. Moreover, LS-102 could attenuate the interaction between SYVN1 and EGFR (Fig. 2G). We also examined the binding of common EGFR mutants (T790M, L858R) to SYVN1 and found that they all could bind to SYVN1 (Fig. 2H). By segmenting EGFR, we determined that SYVN1 interacts with the intracellular domain of EGFR (Fig. S2A, B). Conversely, segmenting SYVN1 revealed that EGFR binds to the N-terminal region (1–290 aa) of SYVN1 (Fig. S2C, D). To ascertain whether the interaction between SYVN1 and EGFR is direct, we purified these two proteins and conducted MBP pull-down experiments, which demonstrated a direct interaction between SYVN1 and EGFR (Fig. 2I). These data indicate that SYVN1 directly interacts with EGFR and that LS-102 can weaken this interaction.

### SYVN1 ubiquitinates EGFR and enhances its stability

**Fig. 3:**
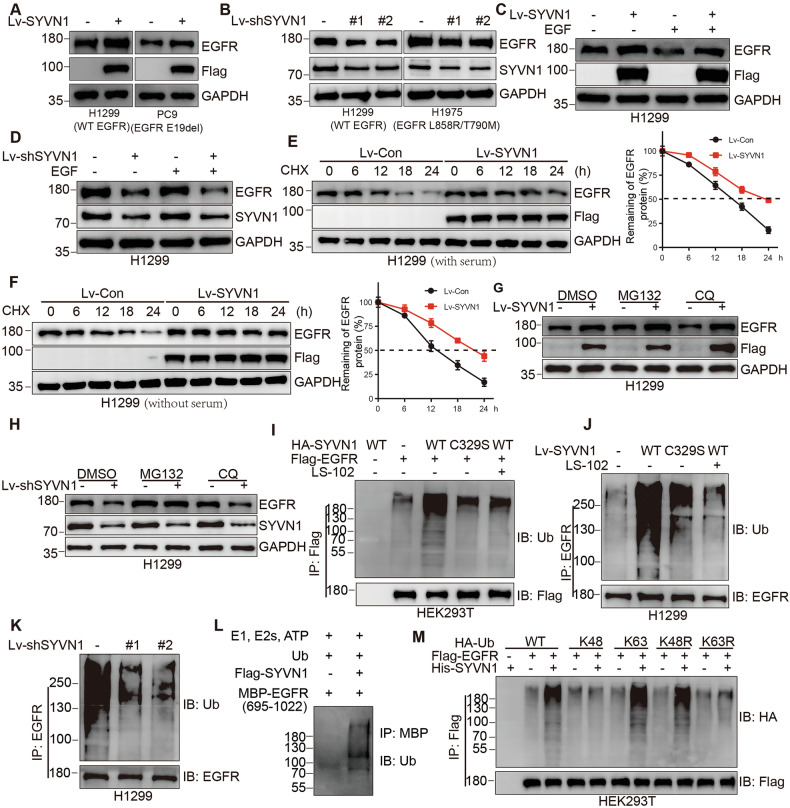
SYVN1 ubiquitinates EGFR and enhances its stability. **A** The EGFR protein was assessed in H1299 and PC9 cells overexpressing SYVN1. **B** The EGFR protein was assessed in H1299 and H1975 cells expressing shSYVN1. **C**, **D** The EGFR protein was detected in H1299 cells with SYVN1 overexpression (**C**) or knockdown (**D**) under conditions with or without EGF (100 ng/mL) stimulation for 10 min. **E**, **F** H1299 cells were treated with CHX (50 μg/mL) for various times, and the degradation rate of EGFR protein in H1299 cells overexpressing SYVN1 under conditions with (**E**) or without (**F**) serum was evaluated. **G**, **H** H1299 cells overexpressing (**G**) or knocking down (**H**) SYVN1 were treated with MG132 (20 μM) or CQ (100 μM) for 6 h, and the EGFR protein levels were detected by Western blot. **I** HEK293T cells were co-transfected with HA-SYVN1 (WT or C329S) and Flag-EGFR plasmids. Immunoprecipitation was performed to enrich Flag-EGFR, followed by assessment of its ubiquitination levels. **J** The EGFR protein was immunoprecipitated in H1299 cells overexpressing SYVN1 (WT or C329S), and its ubiquitination levels were assessed by Western blot. **K** The EGFR protein was immunoprecipitated in H1299 cells with SYVN1 knocked down, and its ubiquitination levels were assessed by Western blot. **L** In vitro ubiquitination assays were performed using purified SYVN1 and EGFR. **M** HEK293T cells were co-transfected with His-SYVN1, Flag-EGFR and various HA-Ub (WT, K48, K63, K48R, or K63R) plasmids. Immunoprecipitation was performed to enrich Flag-EGFR, followed by assessment of its ubiquitination levels by Western blot. All experiments were conducted independently three times. **E**, **F** Data are represented as the mean ± SD. *P* values were determined by independent samples *t* test, *p < 0.05; **p < 0.01; ***p < 0.001.

Next, we investigated the molecular effects of SYVN1 on EGFR. Generally, ubiquitin-related proteins are known to decrease the levels of their substrate proteins [41]. Therefore, we assessed the protein expression of EGFR in NSCLC cell lines with either overexpression or knockdown of SYVN1. Surprisingly, we found that the overexpression of SYVN1 resulted in an increase, rather than a decrease, in the protein levels of EGFR (Fig. 3A). On the contrary, the knockdown of SYVN1 decreased the EGFR protein expression (Fig. 3B). We then explored whether the regulation of EGFR protein expression by SYVN1 was dependent on EGF stimulation. Our results indicated that, regardless of EGF stimulation, overexpression of SYVN1 consistently increased EGFR protein levels, while the knockdown of SYVN1 reduced EGFR protein levels (Fig. 3C, D). Furthermore, we observed that overexpression of SYVN1 significantly prolonged the half-life of EGFR protein, independent of the serum presence (Fig. 3E, F). These data suggest that SYVN1 enhances the stability of EGFR, and this process does not rely on EGF stimulation.

Later, we set out to explore whether SYVN1 acts as a bona fide E3 ubiquitin ligase for EGFR. We found that the proteasome inhibitor MG132 further upregulated the EGFR protein levels induced by SYVN1 overexpression, while the lysosomal inhibitor chloroquine (CQ) did not (Fig. 3G). Similarly, MG132 effectively reversed the decrease in EGFR protein levels caused by SYVN1 knockdown, while CQ could not (Fig. 3H). Furthermore, overexpression of SYVN1 in HEK293T and H1299 cells resulted in an increase in polyubiquitin chains on EGFR. However, the overexpression of the SYVN1 mutant C329S did not achieve this effect, and the compound LS-102 was able to inhibit the increase in polyubiquitin chains induced by SYVN1 (Fig. 3I, J). In contrast, knockdown of SYVN1 led to a reduction in polyubiquitin chains on EGFR (Fig. 3K). In vitro ubiquitination assays using purified SYVN1 and EGFR also demonstrated that SYVN1 increased the polyubiquitin chains on EGFR (Fig. 3L). Importantly, we found that the overexpression of SYVN1 promoted the formation of Lys 63-linked polyubiquitin chains on EGFR, rather than Lys 48-linked chains (Fig. 3M). Therefore, SYVN1 binds to and facilitates the formation of Lys 63-linked polyubiquitin chains on EGFR, thereby inhibiting its proteasomal degradation.

### SYVN1 inhibits the endocytosis of EGFR

**Fig. 4:**
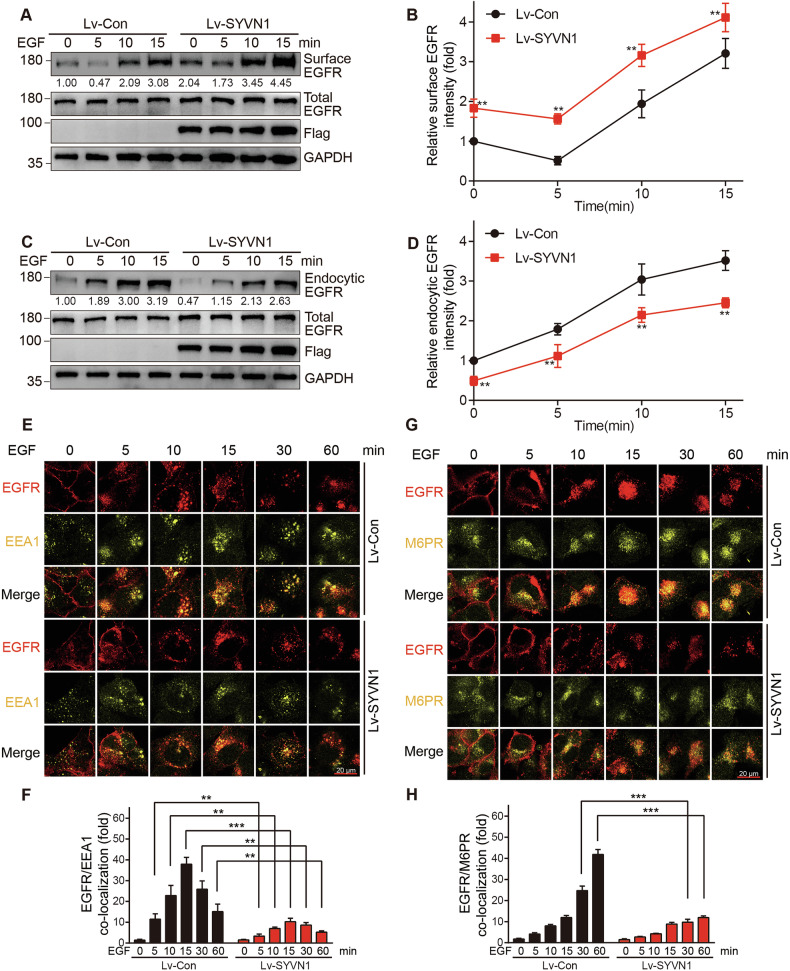
SYVN1 inhibits the endocytosis of EGFR. **A**, **B** Overexpression of SYVN1 increases the membrane localization of EGFR in H1299 cells. H1299 cells were serum-starved overnight and stimulated with 20 ng/mL EGF for varying durations, followed by biotinylation of membrane proteins. Membrane-localized biotinylated EGFR and total EGFR were detected by Western blot. **C**, **D** Overexpression of SYVN1 reduces the cytoplasmic localization of EGFR in H1299 cells. H1299 cells were serum-starved overnight, followed by biotin labeling of membrane proteins, and subsequently stimulated with 20 ng/mL EGF for different time intervals. Cytosolic biotinylated EGFR and total EGFR were detected by Western blot. **E**, **F** Representative images of EGFR and EEA1 co-staining in SYVN1-overexpressing or control cells (**E**) and quantification of EGFR/EEA1 co-localization (**F**). H1299 cells were serum-starved overnight, stimulated with 20 ng/mL EGF for varying time intervals, and then fixed for staining. **G**, **H** Representative images of EGFR and M6PR co-staining in SYVN1 overexpression and control cells (**G**), along with the quantification of EGFR/M6PR co-localization (**H**). H1299 cells were serum-starved overnight, stimulated with 20 ng/mL EGF for varying time intervals, and then fixed for staining. All experiments were conducted independently three times. **B**, **D**, **F**, **H** Data are represented as the mean ± SD. *P* values were determined by independent samples *t* test, *p < 0.05; **p < 0.01; ***p < 0.001. Bars indicate 20 µm.

The signaling of EGFR is tightly regulated by its cellular localization. The binding of the EGF ligand to EGFR activates signaling and initiates a complex cascade that leads to EGFR inactivation through the stimulation of endocytosis. This process is followed by targeting to the lysosome for degradation [42, 43]. Studies have shown that the ubiquitination of the receptor proteins plays a crucial role in their endocytic transport [44]. Therefore, we next examined the effects of SYVN1 on the membrane localization and endocytosis of EGFR. We found that overexpression of SYVN1 increased the levels of EGFR at the cell membrane while decreasing its levels in the cytoplasm (Fig. 4A–D). Conversely, the knockdown of SYVN1 resulted in a decrease in EGFR levels at the cell membrane and an increase in its levels in the cytoplasm (Fig. S3A–D). To further confirm the impact of SYVN1 on EGFR endocytosis, we utilized immunofluorescence staining to track the localization of EGFR in intracellular vesicles. Our results showed that overexpression of SYVN1 reduced the presence of EGFR in both early and late endosomes (Fig. 4E–H), while knockdown of SYVN1 led to an increased presence of EGFR in these endosomes (Fig. S3E–H). In summary, these findings indicate that SYVN1 inhibits the endocytosis of EGFR, leading to its retention at the cell membrane.

### SYVN1 activates the EGFR signaling pathway

**Fig. 5:**
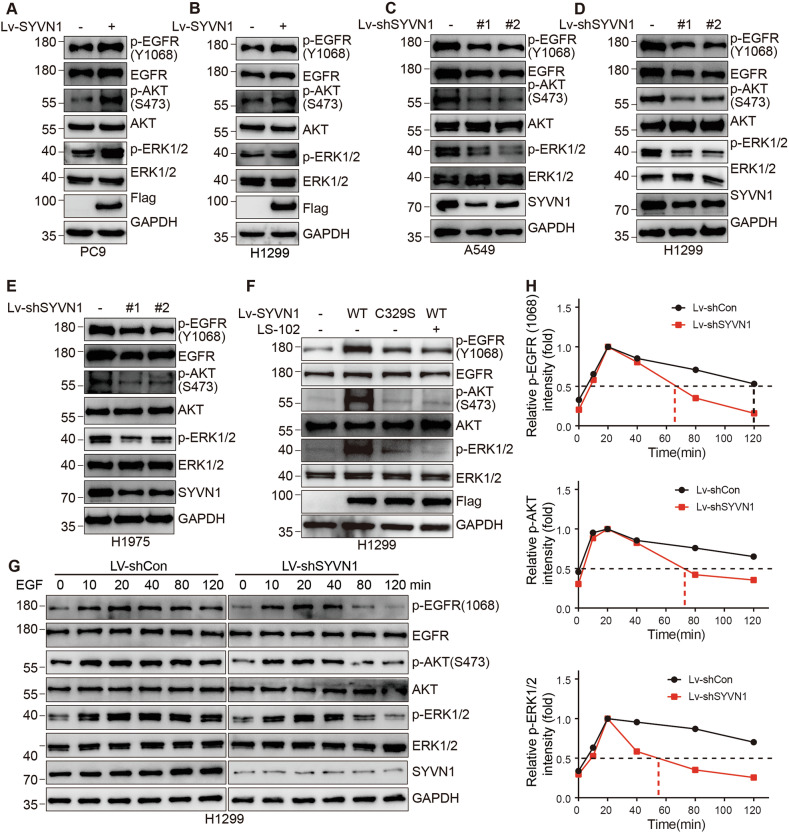
SYVN1 activates the EGFR signaling pathway. **A**, **B** Upregulation of SYVN1 dramatically promoted the activation of EGFR signaling in PC9 (**A**) and H1299 (**B**) cells, as indicated by increased phosphorylation of EGFR, ERK1/2, or AKT. **C**–**E** Downregulation of SYVN1 markedly inhibited the activation of EGFR signaling in A549 (**C**), H1299 (**D**), and H1975 (**E**) cells, as indicated by decreased phosphorylation of EGFR, ERK1/2, or AKT. **F** H1299 cells overexpressing either WT or C329S mutant SYVN1, or treated with LS-102, were subjected to Western blot analysis to assess EGFR signaling activity. **G**, **H** Upregulation of SYVN1 delayed the attenuation of EGFR signaling. H1299 cells overexpressing SYVN1 were starved overnight and then stimulated with EGF (20 ng/mL) for the indicated times. All experiments were conducted independently three times.

The levels of EGFR protein and its cellular localization influence signaling activity. Additionally, the interaction between SYVN1 and EGFR enhances EGFR stability while inhibiting its endocytosis. Therefore, we investigated the effect of SYVN1 on the activity of the EGFR signaling pathway. In agreement with our hypothesis, we observed that the overexpression of SYVN1 in PC9 and H1299 cells resulted in a significant increase in the phosphorylation levels of EGFR, AKT, and ERK1/2 (Fig. 5A, B). Conversely, the knockdown of SYVN1 in A549, H1299, and H1975 cells led to a reduction in the phosphorylation levels of EGFR, AKT, and ERK1/2 (Fig. 5C–E). In H1299 cells, overexpression of SYVN1 enhanced the phosphorylation of EGFR, AKT, and ERK1/2, but the C329S mutant did not exhibit this effect. Additionally, LS-102 was able to weaken the effects of EGFR activation (Fig. 5F). More importantly, we found that knockdown of SYVN1 resulted in a faster attenuation of EGFR signaling activity, which is detrimental to the rapid proliferation of tumor cells (Fig. 5G, H).

### The expression of SYVN1 in NSCLC tissues is positively correlated with EGFR and negatively associated with prognosis

**Fig. 6:**
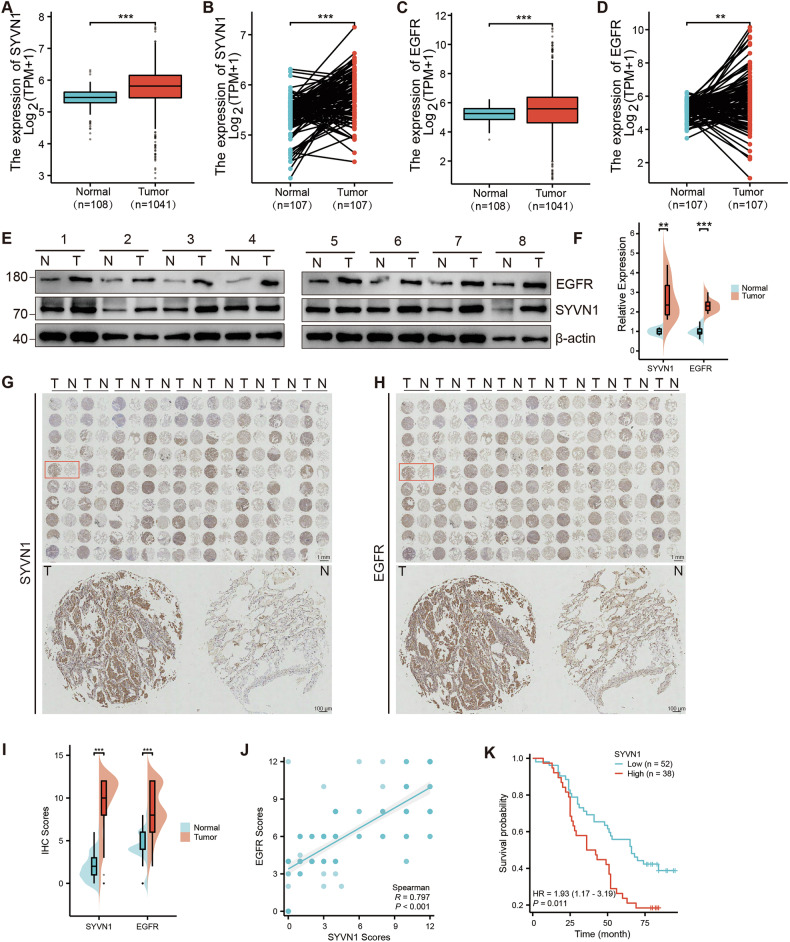
The expression of SYVN1 in NSCLC tissues is positively correlated with EGFR and negatively associated with prognosis. **A**–**D** The mRNA levels of SYVN1 (**A**, **B**) and EGFR (**C**, **D**) in NSCLC and normal lung tissues were analyzed through the TCGA database. **E**, **F** SYVN1 and EGFR proteins are highly expressed in NSCLC tissues compared to normal tissues. **G**, **H** IHC staining analysis of SYVN1 and EGFR was performed in NSCLC tissue microarrays, and representative IHC staining images are shown. **I** The IHC scores of SYVN1 and EGFR in (**G**, **H**). **J** The correlation analysis was conducted between SYVN1 and EGFR scores using Spearman’s correlation coefficient. **K** Kaplan−Meier plots of overall survival (OS) for patients with tissue microarrays.

SYVN1 enhances the stability of EGFR and increases its protein levels. We aimed to investigate the relationship between their expressions in clinical NSCLC tissues. Initially, the mRNA expression levels of SYVN1 and EGFR in the NSCLC sequencing data obtained from The Cancer Genome Atlas (TCGA) database (the TCGA Pan-Cancer Clinical Data Resource) were analyzed using the Xiantao Academic Bioinformatics Analysis Platform (https://www.xiantaozi.com/). In both paired and unpaired analyses, we observed that the expression levels of SYVN1 and EGFR were significantly higher in NSCLC tissues than in normal tissues (Fig. 6A–D). Next, we detected the protein expression levels of SYVN1 and EGFR, as well as the correlation between SYVN1 expression and the progression of NSCLC in pairs of tumor specimens. Both SYVN1 and EGFR were expressed at higher levels in tumor tissues compared to normal tissues, as determined by Western blot (Fig. 6E, F). Moreover, immunohistochemical staining was also conducted to further analyze the protein expression levels of SYVN1 and EGFR. Similarly, in our collected tumor tissues, we observed high expression levels of SYVN1 and EGFR in tumor tissues and a positive correlation between their expression (Fig. S4A–C). In addition, in the NSCLC tissue microarray (90 pairs), we also found that SYVN1 and EGFR were expressed at higher levels, and their expressions were positively correlated (Fig. 6G–J) (Table S1). Subsequently, we analyzed the correlation between SYVN1 expression and the clinicopathological characteristics of NSCLC patients. Our findings indicate that high expression of SYVN1 was strongly associated with OS event (Table S2). Furthermore, we consistently observed that SYVN1 expression correlated with overall survival in patients with NSCLC based on univariate and multivariate survival analyses (Table S3), as did TNM stage. Finally, Kaplan-Meier analysis was performed on the Xiantao platform to assess the correlation between the expression levels of SYVN1 and EGFR and patient survival rates. As shown in Fig. 6K and Fig. S4D and E, univariate analysis of overall survival in NSCLC samples revealed that higher expression levels of SYVN1 and EGFR are associated with poorer patient prognosis. Taken together, these findings suggest that high expression levels of SYVN1 contribute to the progression of NSCLC.

### LS-102 overcomes resistance to AZD9291 in NSCLC cells

Resistance to EGFR tyrosine kinase inhibitors (EGFR-TKIs) poses a significant challenge in the treatment of NSCLC. The UPR pathway plays an important role in chemoresistance [45, 46]. As an important regulatory protein in UPR, we hypothesized that the interaction between SYVN1 and EGFR is related to chemoresistance. To test this hypothesis, we first examined the activation of the UPR pathway. We found that the overexpression of SYVN1 in H1299 cells slightly activated the UPR (PERK, IRE1α and ATF6) pathway, while SYVN1 significantly activated the UPR pathway under AZD9291 treatment, which was inhibited by overexpression of C329S (Fig. S5A). Then, the interaction between SYVN1 and EGFR in H1299 cells treated with AZD9291 was detected, and it was found that AZD9291 enhanced their interaction (Fig. S5B). In addition, SYVN1 overexpression inhibited apoptosis and promoted cell proliferation and colony formation in response to AZD9291 treatment, as demonstrated by apoptotic protein detection, the CCK-8 assay, and the colony formation assay. However, the C329S mutation attenuated these effects (Fig. S5C–L). Furthermore, the inhibition of the UPR pathway by the PERK inhibitor (GSK2606414) or the IRE1α inhibitor (4μ8C), as well as the knockdown of PERK or IRE1α, inhibited SYVN1-suppressed apoptosis and promoted proliferative activities (Fig. S5G–L). In contrast, the overexpression of PERK in C329S-overexpressing cells inhibited cell apoptosis and promoted cell proliferation (Fig. S5G–L). These data suggest that the interaction between SYVN1 and EGFR plays an important role in the activation of the UPR pathway and drug resistance.

**Fig. 7:**
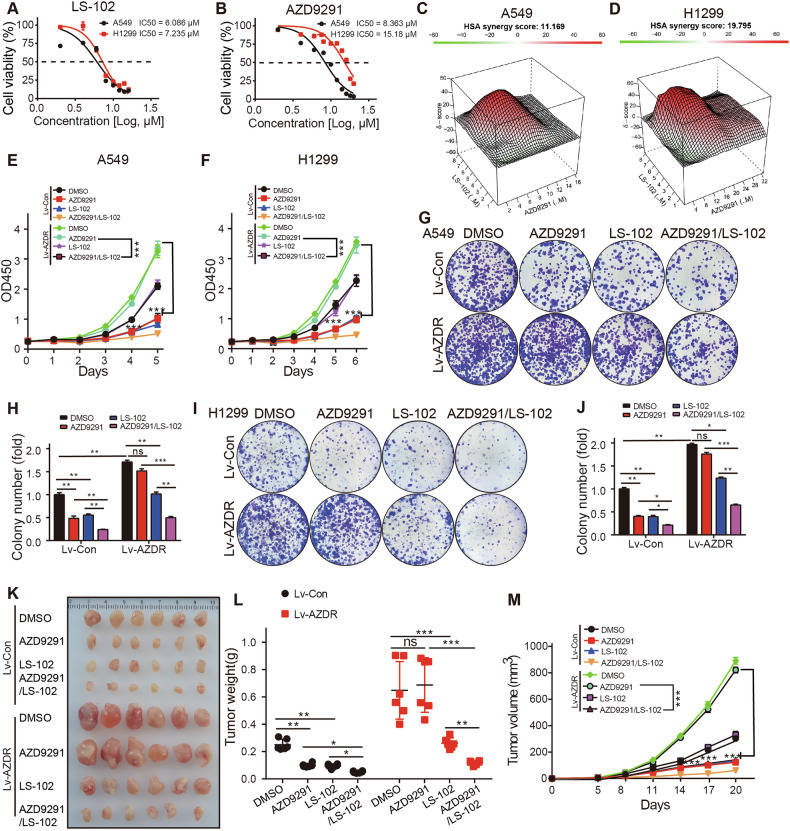
LS-102 overcomes resistance to AZD9291 in NSCLC cells. **A**, **B** Varying responses of A549 and H1299 cells to LS-102 (**A**) or AZD9291 (**B**) treatment. **C**, **D** The synergistic effects of LS-102 and AZD9291 were assessed using the SynergyFinder tool in A549 (**C**) and H1299 (**D**) cells. **E** A549 cells overexpressing EGFR^T790M/C797S^ were treated with either LS-102 (5 μM), AZD9291 (6 μM), or both, and cell proliferation was evaluated using CCK-8. **F** H1299 cells overexpressing EGFR^T790M/C797S^ were treated with either LS-102 (5 μM), AZD9291 (8 μM), or both, and cell proliferation was evaluated using CCK-8. **G**, **H** A549 cells overexpressing EGFR^T790M/C797S^ were treated with either LS-102 (5 μM), AZD9291 (6 μM), or both, and cell colony formation assays were performed. **I**, **J** H1299 cells overexpressing EGFR^T790M/C797S^ were treated with either LS-102 (5 μM), AZD9291 (8 μM), or both, and cell colony formation assays were performed. **K**–**M** H1299 cells overexpressing EGFR^T790M/C797S^ were implanted subcutaneously into nude mice. The mice were administered either AZD9291 (5 mg/kg, via daily gastric gavage), LS-102 (3 mg/kg, via daily intraperitoneal injection), or both for 3 weeks. At the experimental endpoint, tumors were excised, photographed (**K**), and weighed (**L**). The tumor growth curves were then generated (**M**). **A**, **B**, **E**, **F**, **H**, **J**, **L**, **M** Data were presented as mean ± SD. *P* values were determined by independent samples *t-*test, *p < 0.05; **p < 0.01; ***p < 0.001.

Currently, no new EGFR-TKIs have been approved for AZD9291-resistant cases. Our previous data indicated that LS-102 effectively inhibits NSCLC cell proliferation. Therefore, we investigated the effects of LS-102 on NSCLC cells resistant to AZD9291. We first assessed cell viability in A549 and H1299 cells treated with varying concentrations of LS-102 or AZD9291 and calculated the IC50 values (Fig. 7A, B). Based on IC50 values, we selected two concentrations of LS-102 (5 μM and 10 μM) to treat H1299 cells. Western blot analysis was conducted to assess the changes in SYVN1 and EGFR protein levels. Our results showed that SYVN1 levels remained unaffected at both concentrations, while EGFR protein levels significantly decreased (Fig. S6A). Besides, treatment with LS-102 accelerated the degradation rate of EGFR protein in H1299 cells (Fig. S6B). Furthermore, we found that LS-102 can inhibit the stabilizing effect of SYVN1 overexpression on EGFR protein, thereby accelerating its degradation (Fig. S6C, D). These data indicate that LS-102 exerts its effects by inhibiting the enzymatic activity of SYVN1 without affecting its protein levels.

Then, a series of concentration ratios for LS-102 and AZD9291 were established to treat H1299 and A549 cells, and the cell viability was measured using CCK-8. We then utilized the computational tool SynergyFinder (https://synergyfinder.fimm.fi) to evaluate whether these compounds exhibited any synergistic effects, as this tool is specifically designed for calculating the combinatory effects of two drugs [47]. Computational modeling revealed a high synergy score of 11.169 in A549 cells and 19.795 in H1299 cells (Fig. 7C, D). Subsequently, we established A549 and H1299 cell lines that stably overexpress EGFR^T790M/C797S^ to mimic resistance to AZD9291 (AZDR) [48]. By adopting CCK-8 assays, we found that the combination of LS-102 and AZD9291 synergistically inhibited the proliferation of A549 and H1299 cells, with LS-102 effectively overcoming cellular resistance to AZD9291 in AZDR cells (Fig. 7E, F). Similar results were obtained from colony formation assays (Fig. 7G–J). Furthermore, in H1299/AZDR xenograft mouse models, the combination of LS-102 and AZD9291 significantly suppressed the growth of NSCLC, and LS-102 overcame resistance to AZD9291 in AZDR cells (Fig. 7K–M). Together, these results indicate that the combined use of LS-102 and AZD9291 can effectively overcome AZD9291 resistance induced by EGFR^T790M/C797S^ in NSCLC. This may provide a new direction for the treatment of patients with AZD9291 resistance.

## Discussion

NSCLC remains a leading cause of cancer-related mortality, and the emergence of resistance to EGFR-TKIs like AZD9291 significantly complicates treatment strategies [6]. Our study identifies SYVN1 as a novel therapeutic target in NSCLC, particularly in the context of TKI resistance. The findings suggest that targeting the SYVN1-EGFR axis may provide a promising strategy to enhance the efficacy of existing therapies and overcome resistance mechanisms.

Numerous studies have shown that the interaction between E3 ubiquitin ligases and substrate proteins leads to the ubiquitination and subsequent degradation of these substrates. This process influences the progression of various diseases, including cancer [48–50]. The role of E3 ubiquitin ligases is determined by the substrates they regulate. The degradation of oncogenic proteins by certain E3 ligases can inhibit tumor progression [48, 51–53], while the degradation of tumor suppressor proteins may promote tumorigenesis [26, 54–56]. Moreover, even the same ligase may exhibit different effects in various environments. Our results demonstrate that SYVN1 directly interacts with EGFR, facilitating its stabilization through Lys 63-linked polyubiquitination, thereby inhibiting proteasomal degradation. This result contrasts with the majority of existing studies focused on substrate degradation [48, 57]. This mechanism contributes to sustained EGFR signaling, promoting tumor growth both in vitro and in vivo. The elevated expression of SYVN1 in NSCLC correlates with poor patient prognosis, underscoring its potential as a biomarker and therapeutic target. Importantly, the inhibition of SYVN1 by LS-102, a selective inhibitor of SYVN1, not only reduces EGFR levels but also diminishes its signaling activity, highlighting the dual role of SYVN1 in both stabilizing EGFR and enhancing its signaling.

Multiple studies have highlighted the significance of EGFR internalization via endocytosis in the regulation of EGFR activity [15, 58]. Our data indicate that SYVN1 inhibits the endocytosis of EGFR, leading to increased receptor availability at the cell membrane (Fig. S7). This retention is critical for sustaining the activation of downstream signaling pathways, such as AKT and ERK1/2, which are pivotal for cell proliferation and survival. The ability of SYVN1 to modulate EGFR localization and activity presents a unique mechanism by which cancer cells can evade therapeutic interventions. The unfolded protein response (UPR) and ERAD pathways play key roles in chemoresistance [45, 46]. As an important regulatory protein in these pathways, we found that SYVN1 activates UPR pathways (PERK, IRE1α, and ATF6) to mediate chemoresistance. In the tumor microenvironment, the UPR and the AKT/ERK pathway are often activated together, promoting cancer cells’ ability to adapt to stress, resist apoptosis, and enhance drug resistance [59–62]. It was reported that EGFR triggers endoplasmic reticulum stress and activates the UPR pathway through downstream signaling pathways such as RAS/MAPK and PI3K/AKT, inhibiting cell apoptosis and promoting tumor cell survival and drug resistance [63]. In turn, the activation of the UPR regulates EGFR expression and activity [64]. Molecular chaperones upregulated by the UPR, such as GRP78, assist EGFR in folding correctly or stabilizing its conformation, which affects its membrane localization and signaling activity [65]. Additionally, effector molecules of the UPR, such as ATF4 and XBP1, may directly or indirectly regulate EGFR gene expression and influence its downstream signaling pathways. However, when EGFR signaling is hyperactivated, the UPR also degrades EGFR via ERAD or autophagy pathways. Taken together, the UPR cross-talks with the AKT/ERK pathway through the PERK, IRE1, and ATF6 branches, thereby co-regulating cell survival, metabolism, and stress adaptation. The interaction of these pathways is not only an important mechanism of tumor drug resistance but also provides a theoretical basis for the development of combined treatment strategies.

Targeting the degradation of the EGFR protein demonstrates a significant inhibitory effect on tumor growth, potentially offering a new direction for the treatment of NSCLC [4, 48]. The combination of EGFR-TKIs with other targeted molecular inhibitors has demonstrated promising efficacy in overcoming resistance to EGFR-TKIs [66, 67]. We found that the combination of LS-102 with AZD9291 demonstrates a synergistic effect, effectively overcoming AZD9291 resistance in NSCLC cell lines and xenograft models. This finding is particularly significant given the limited options currently available for patients harboring mutations such as EGFR^T790M^ and EGFR^C797S^. By destabilizing EGFR through SYVN1 inhibition, LS-102 re-sensitizes NSCLC cells to EGFR-TKI treatment, providing a potential pathway for improving patient outcomes. These data indicate that LS-102 has the potential to be an innovative therapeutic option for a variety of diseases regulated by SYVN1. However, it is worth noting that although LS-102 has shown potential as a targeted therapeutic agent in preclinical models, there are still limitations and concerns [68, 69]. Firstly, as a SYVN1-specific inhibitor, LS-102 may disrupt the ubiquitin-proteasome system, potentially interfering with the metabolism of other drugs that rely on this system for degradation. Additionally, this system plays a key role in immune regulation, which may affect immune cell function. Secondly, inhibiting SYVN1 may increase ER stress and disrupt protein homeostasis in metabolically active organs such as the liver and kidneys, potentially leading to elevated liver enzymes or abnormal renal function. Thirdly, while studies have shown that LS-102 has an anti-inflammatory effect, excessive inhibition of the inflammatory response could increase the risk of secondary infections or impair wound healing. Fourth, the pharmacokinetics and bioavailability of LS-102 require further optimization to ensure effective delivery and minimize toxicity. Finally, another limitation lies in the possibility that the development of resistance may occur, as cancer cells may adapt by activating compensatory pathways. Therefore, a comprehensive evaluation of the long-term toxicity and resistance mechanisms of LS-102 is essential before advancing it into clinical use.

Existing studies established EGFR as a key driver in NSCLC and highlighted the challenge of acquired resistance to EGFR-TKIs. Previous studies have primarily focused on downstream signaling pathways, secondary mutations, and combination therapies targeting parallel pathways to overcome this resistance. However, the specific role of E3 ubiquitin ligases, such as SYVN1, in regulating EGFR stability and signaling has been less thoroughly explored. Our study advances this field by identifying SYVN1 as a novel regulator of EGFR in NSCLC. Unlike prior work, we demonstrate that SYVN1 directly interacts with the intracellular domain of EGFR via its N-terminus (1–290 aa), promoting Lys 63-linked ubiquitination, which inhibits both proteasomal degradation and endocytosis of EGFR. This stabilization leads to enhanced EGFR signaling, contributing to tumor growth and resistance. Importantly, we also show that LS-102 can destabilize EGFR, counteract SYVN1-mediated stabilization, and synergize with AZD9291 to reverse resistance. The focus on the SYVN1-EGFR axis is novel and shifts the research paradigm from targeting downstream pathways to the direct regulation of EGFR stability through ubiquitination. In the context of EGFR-TKI resistance, especially for mutations such as T790M, C797S, and other mechanisms that maintain EGFR signaling despite the use of TKIs, our results provide a new therapeutic strategy to overcome resistance by destabilizing EGFR through the inhibition of SYVN1. Thus, our study fills an important gap by elucidating a previously underestimated mechanism of EGFR post-translational regulation and proposing targeted EGFR degradation as a means to improve therapeutic outcomes in drug-resistant NSCLC.

Imperfectly, our study did not elucidate the mechanism by which SYVN1 inhibits the endocytosis of the EGFR. It remains unclear whether SYVN1 functions as a scaffold protein that inhibits the recruitment of partner proteins, such as Eps15 and Epsin1 [70, 71], to EGFR. Besides, the effect of SYVN1 on the recycling of EGFR remains unknown. Furthermore, while current studies on SYVN1 mainly focus on its ubiquitination, future research should explore its association with other post-translational modifications, especially how these may modulate the role of SYVN1 in ER stress. In addition, further studies are needed to link the multiple mechanisms by which SYVN1 functions in normal and pathological conditions. This includes elucidating how SYVN1 operates differently in various tumors and its dual roles in processes such as metastasis and invasion within the same tumor. These insights will help optimize therapeutic interventions for various clinical conditions. Finally, the specific roles and mechanisms of SYVN1 in various modes of cell death (such as pyroptosis and ferroptosis) and biological processes (such as autophagy and the cell cycle) urgently need to be explored to provide new insights into the regulation of these processes. Future research should advance in these directions and further explore the functional role and mechanisms of SYVN1 in various physiological diseases. This will enhance our understanding of SYVN1 and provide more accurate and effective treatment methods for different diseases.

## Supplementary information

Supplementary Figure Legends

Supplementary Figure S1

Supplementary Figure S2

Supplementary Figure S3

Supplementary Figure S4

Supplementary Figure S5

Supplementary Figure S6

Supplementary Figure S7

Table S1–3

Western Blots

## Data Availability

The datasets generated in this study are available from the corresponding author upon reasonable request and will be provided in accordance with institutional guidelines.
