# Supplementary material for: Targeting the SYVN1-EGFR axis: a breakthrough strategy for TKI-resistant NSCLC
Source: Cell Death Dis. 2025 Aug 28;16(1):655. doi: 10.1038/s41419-025-07978-2 (PMC12394631; doi:10.1038/s41419-025-07978-2)
Supplement: Supplementary file 1 — Supplementary Figure Legends [file 41419_2025_7978_MOESM1_ESM.docx]

**Supplementary Information**

**Targeting the SYVN1-EGFR Axis: A Breakthrough Strategy for TKI-Resistant NSCLC**

Xinsheng Xie^1, 2, 3#^, Weilai Tong^3^^, 4#^, Yue Xie^5#^, Haoxin Jiang^3, 4#^, Alan Jiang^3^, Junming Huang^3, 4^, Zhili Liu^1, 3, 4*^, Jingjing Yu^6*^

^1^Department of Orthopedic Surgery, The Third Affiliated Hospital, Jiangxi Medical College, Nanchang University, Nanchang, 330006, China.

^2^School of Information Management, Jiangxi University of Finance and Economics, Nanchang, 330006, China.

^3^Jiangxi Provincial Key Laboratory of Spine and Spinal Cord Diseases, Nanchang, 330006, China.

^4^Department of Orthopedic Surgery, The First Affiliated Hospital, Jiangxi Medical College, Nanchang University, Nanchang, 330006, China.

^5^Department of Urology, The First Affiliated Hospital, Jiangxi Medical College, Nanchang University, Nanchang, 330006, China.

^6^Department of Respiratory and Critical Care Medicine, Jiangxi Provincial Key Laboratory of Respiratory Diseases, Jiangxi Institute of Respiratory Diseases, Jiangxi Clinical Research Center for Respiratory Diseases, The First Affiliated Hospital, Jiangxi Medical College, Nanchang University, Nanchang 330006, China.

**^#^These authors contributed equally.**

**^*^Corresponding Authors:** Jingjing Yu, email: [ndyfy01237@ncu.edu.cn;](mailto:ndyfy01237@ncu.edu.cn;) Zhili Liu, email: [zhili-liu@ncu.edu.cn](mailto:zhili-liu@ncu.edu.cn).

**Supplementary Results**

**Supplementary Fig. S1 Efficiency verification of SYVN1 overexpression and knockdown.** (A) Detection of SYVN1 protein expression levels in various NSCLC cell lines by Western blot. (B) Successful overexpression of *SYVN1* in H1299 and PC9 cells infected with a lentivirus expressing *SYVN1* were confirmed by Western blot. The overexpressed SYVN1 is fused with a Flag tag. (C) H1299, H1975 and A549 cells were infected with a lentivirus targeting *SYVN1*, the knockdown efficiency was confirmed by Western blot.

**Supplementary Fig. S2 SYVN1 interacts with EGFR.** (A) Diagrammatic representation of EGFR and various truncated mutants used for mapping the SYVN1-binding domain. (B) Co-IP assays were conducted to map the SYVN1-binding domain of EGFR. (C) Diagrammatic representation of SYVN1 and various truncated mutants used for mapping the EGFR-binding domain. (D) Co-IP assays were conducted to map the EGFR-binding domain of SYVN1.

**Supplementary Fig. S3 Knockdown of SYVN1 promotes EGFR internalization.** (A, B) Knockdown of SYVN1 reduces the membrane localization of EGFR in H1299 cells. H1299 cells were serum-starved overnight and stimulated with 20 ng/mL EGF for varying durations, followed by biotinylation of membrane proteins. Membrane-located biotinylated EGFR and total EGFR were detected by Western blot. (C, D) Knockdown of SYVN1 increases the cytoplasmic localization of EGFR in H1299 cells. H1299 cells were serum-starved overnight, followed by biotin labeling of membrane proteins, and subsequently stimulated with 20 ng/mL EGF for different time intervals. Cytosolic-located biotinylated EGFR and total EGFR were detected by Western blot. (E, F) Representative images of EGFR and EEA1 co-staining in SYVN1 knockdown or control cells (E) and quantification of EGFR/EEA1 co-localization (F). H1299 cells were serum-starved overnight, stimulated with 20 ng/mL EGF for varying time intervals, and then fixed for staining. (G, H) Representative images of EGFR and M6PR co-staining in SYVN1 knockdown or control cells (G) and quantification of EGFR/M6PR co-localization (H). H1299 cells were serum-starved overnight, stimulated with 20 ng/mL EGF for varying time intervals, and then fixed for staining. Data are shown as the mean ± SD. *P* values were determined by independent-samples *t-*test, **p* < 0.05; ***p* < 0.01; ****p* < 0.001. Bars indicate 20 µm.

**Supplementary Fig. S4 SYVN1 and EGFR are highly expressed in NSCLC tissues and positively correlated with poor prognosis.** (A, B) SYVN1 and EGFR are highly expressed in NSCLC tissues compared to paired normal tissues. (C) The expression of SYVN1 and EGFR are positively correlated. (D, E) The expression of SYVN1 and EGFR was positively correlated with poor prognosis in TCGA database. Data were presented as mean ± SD. *P* values were determined by independent-samples *t-*test, **p* < 0.05; ***p* < 0.01; ****p* < 0.001.

**Supplementary Fig. S5 The interaction between SYVN1 and EGFR plays an important role in the activation of the UPR pathway and drug resistance.** (A) Overexpression of SYVN1 activated the UPR pathway, C329S mutant inhibited this effect. (B) AZD9291 enhanced the interaction between SYVN1 and EGFR. (C, D) IC50 values of GSK2606414 were determined in H1299 and PC9 cells. (E, F) IC50 values of 4μ8C were determined in H1299 and PC9 cells. (G) H1299 cells overexpressing SYVN1 were treated with UPR pathway inhibitors (25 nM GSK2606414 for PERK and 30 μM 4μ8C for IRE1α, 24 h) or with siRNA (PERK and IRE1α), and the changes in apoptotic proteins were detected by Western blot. (H) IC50 values of AZD9291 were determined in PC9 cells. (I, K, L) H1299 cells overexpressing SYVN1 were treated with the UPR pathway inhibitors (10 nM GSK2606414 for PERK and 8 μM 4μ8C for IRE1α) or with siRNA (PERK and IRE1α), cell proliferation and colony formation were assessed. (J, K, L) PC9 cells overexpressing SYVN1 were treated with the UPR pathway inhibitors (5 nM GSK2606414 for PERK and 7 μM 4μ8C for IRE1α) or with siRNA (PERK and IRE1α), cell proliferation and colony formation were assessed. Data were presented as mean ± SD. *P* values were determined by independent-samples *t-*test, **p* < 0.05; ***p* < 0.01; ****p* < 0.001.

**Supplementary Fig. S6 LS-102 inhibits the stabilizing effect of SYVN1 on EGFR protein.** (A, B) LS-102 promotes the degradation of EGFR without affecting SYVN1 expression. (C, D) LS-102 inhibits the stabilizing effect of SYVN1 on EGFR protein. Data were presented as mean ± SD. *P* values were determined by independent-samples *t-*test, **p* < 0.05; ***p* < 0.01; ****p* < 0.001.

**Supplementary Fig. S7 Proposed model for SYVN1 promotion of NSCLC growth via activating EGFR signaling.** In tumor cells, SYVN1 directly interacts with EGFR, leading to K63-linked ubiquitination that enhances EGFR stability and inhibits its endocytosis. This process subsequently activates the EGFR signaling pathway, promoting tumor growth. The figure was created with Figdraw.com.
