# Supplementary figures and images for: Targeting the SYVN1-EGFR axis: a breakthrough strategy for TKI-resistant NSCLC
Source: Cell Death Dis. 2025 Aug 28;16(1):655. doi: 10.1038/s41419-025-07978-2 (PMC12394631; doi:10.1038/s41419-025-07978-2)

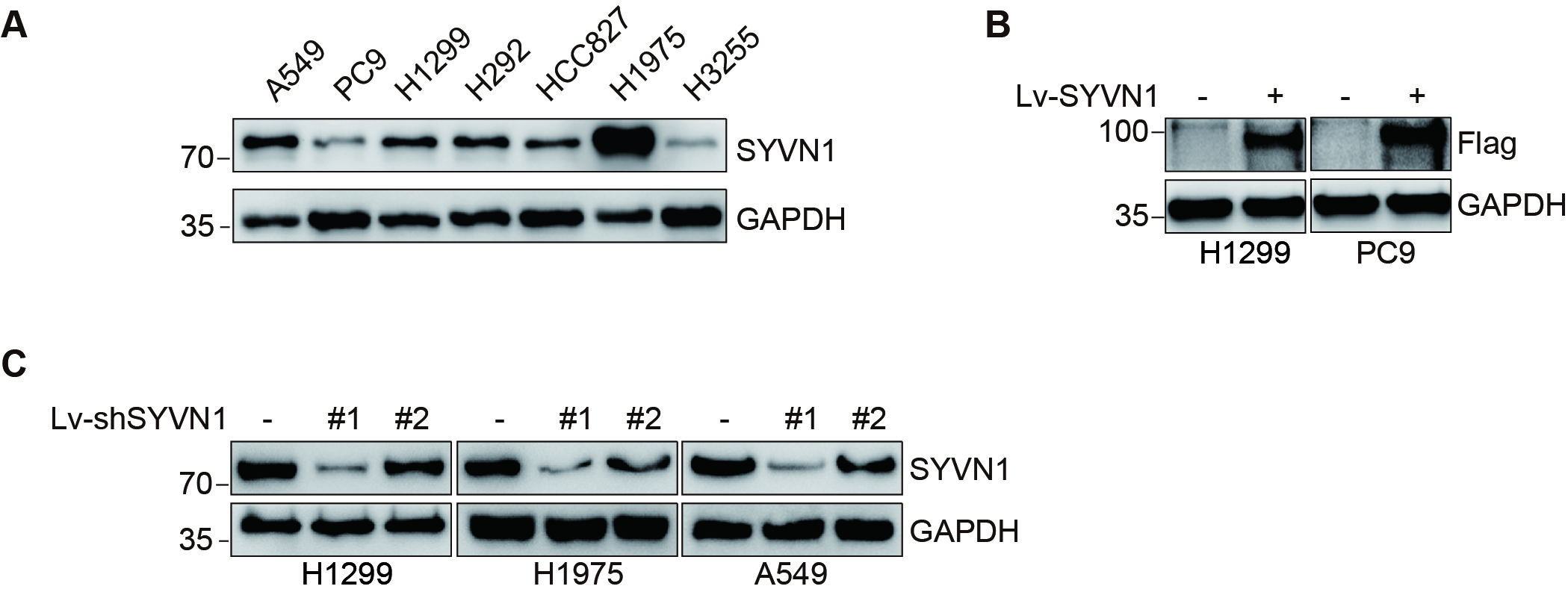

Supplement: Supplementary file 2 — Supplementary Figure S1 [file 41419_2025_7978_MOESM2_ESM.jpg]

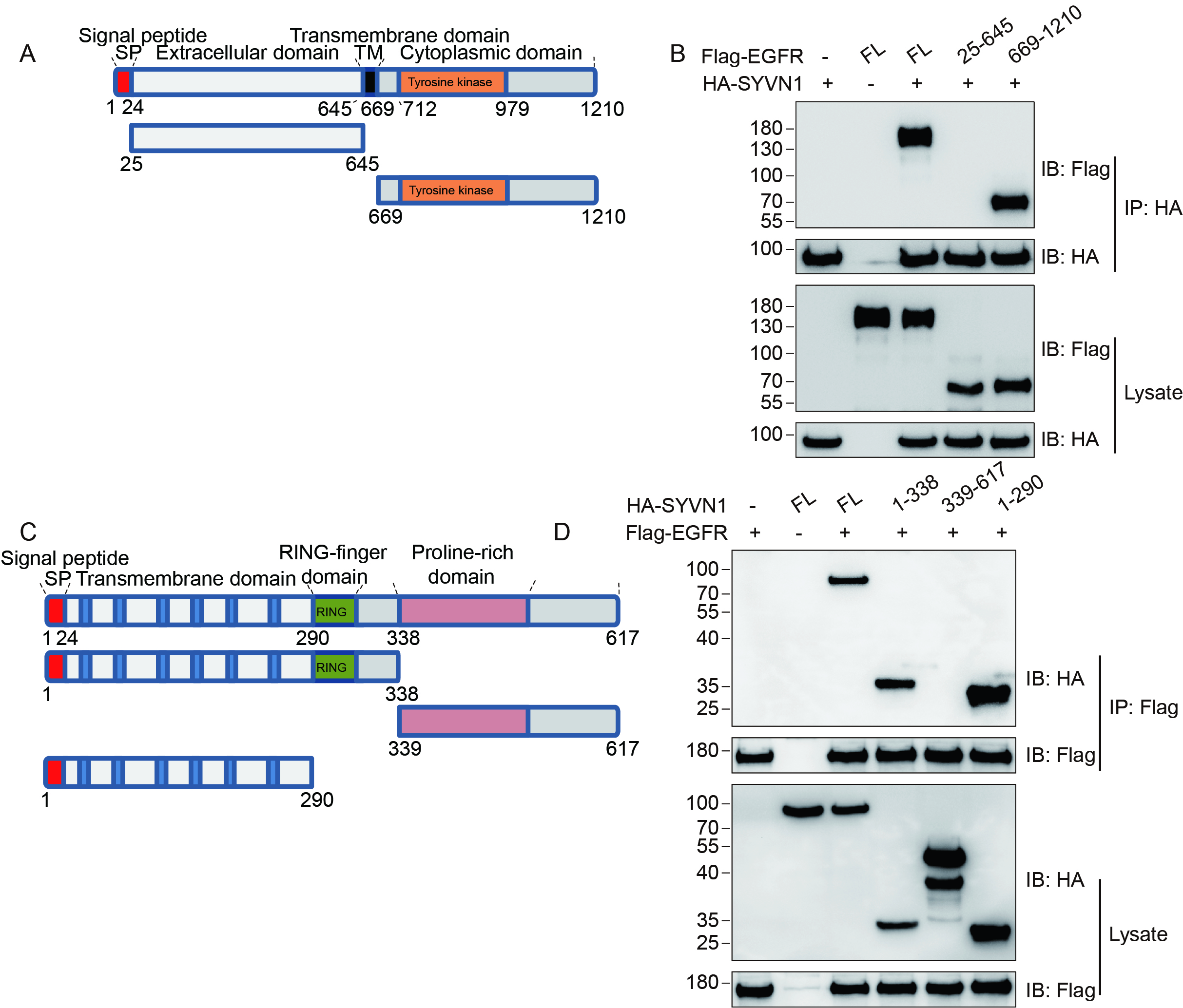

Supplement: Supplementary file 3 — Supplementary Figure S2 [file 41419_2025_7978_MOESM3_ESM.jpg]

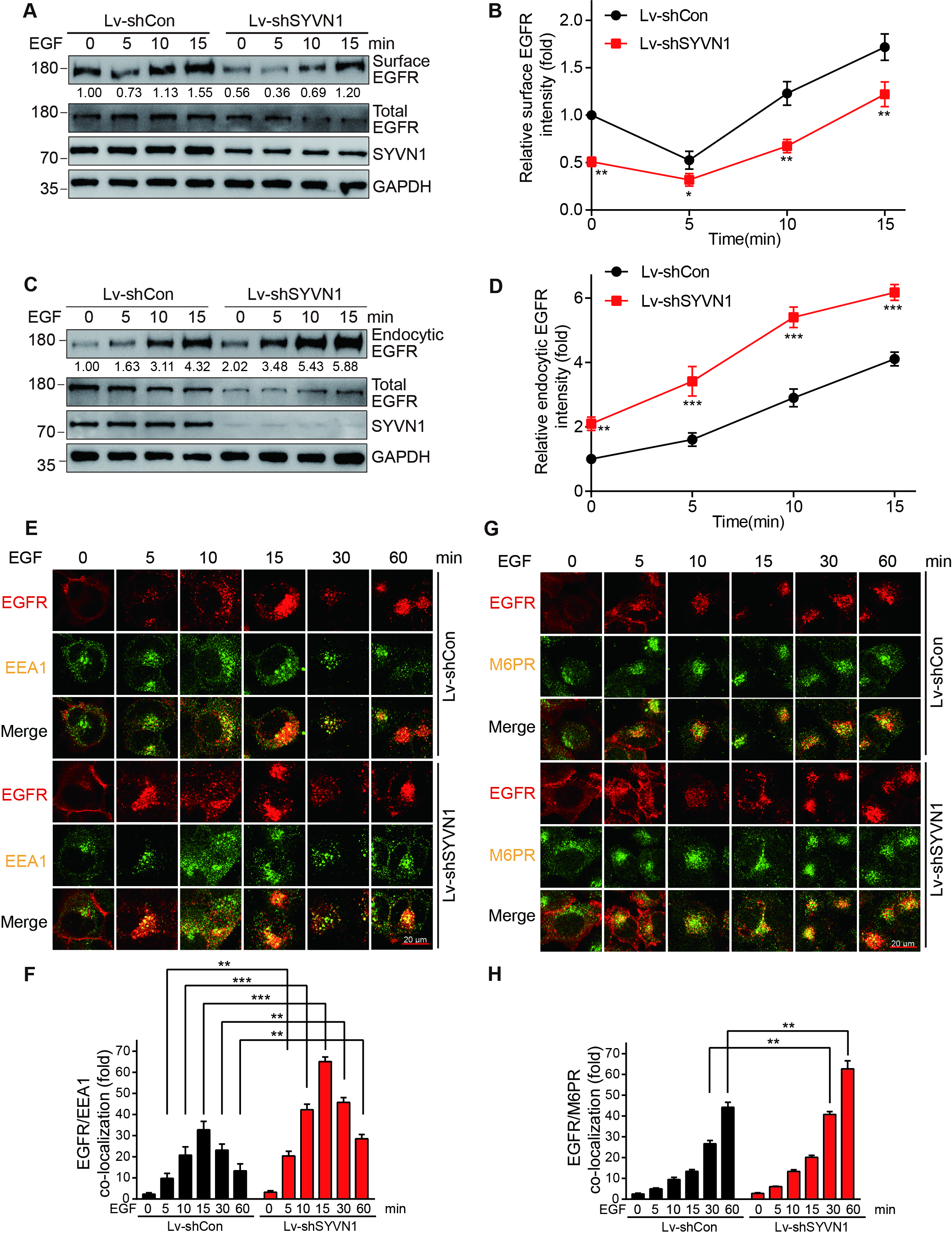

Supplement: Supplementary file 4 — Supplementary Figure S3 [file 41419_2025_7978_MOESM4_ESM.jpg]

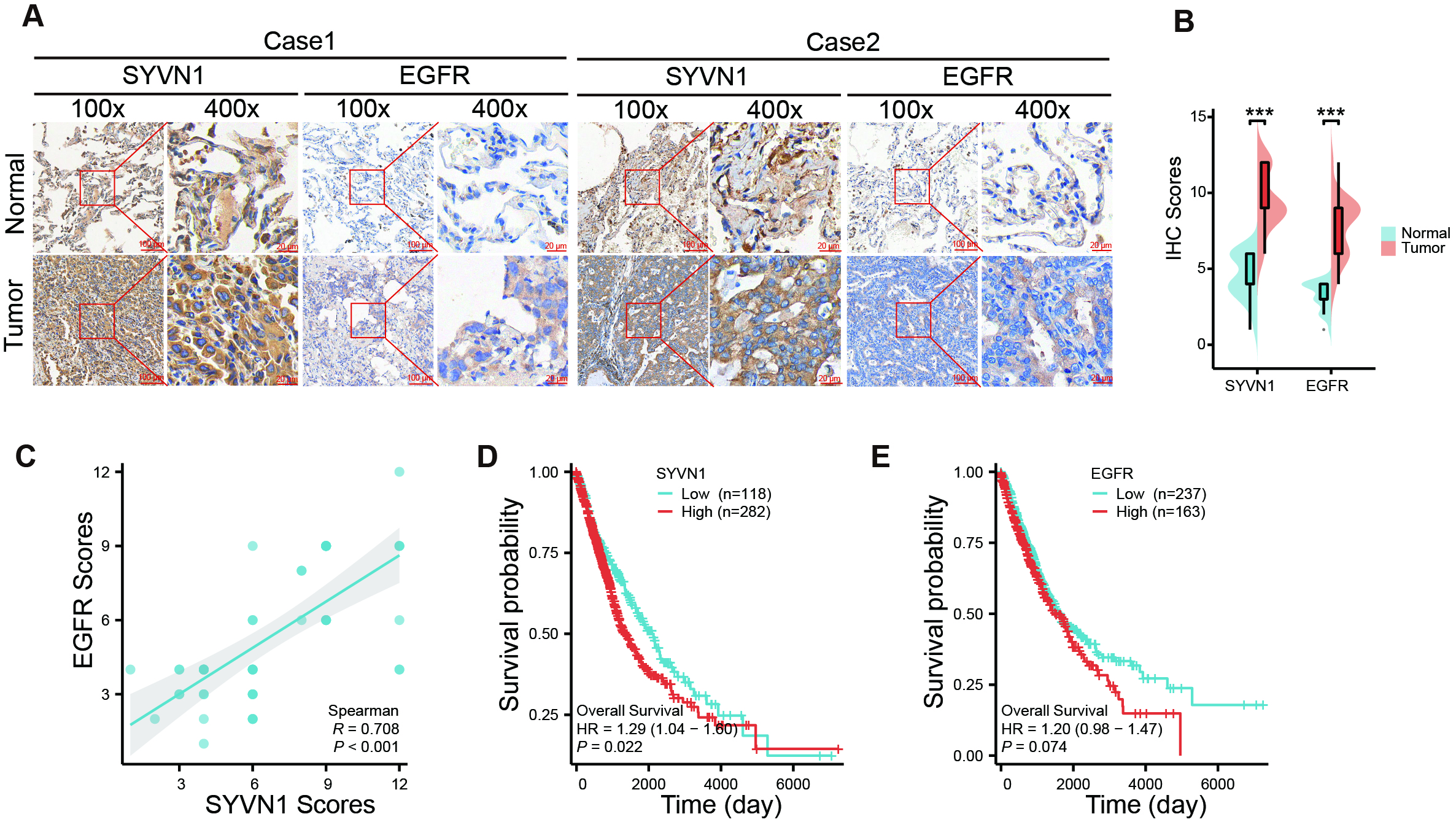

Supplement: Supplementary file 5 — Supplementary Figure S4 [file 41419_2025_7978_MOESM5_ESM.jpg]

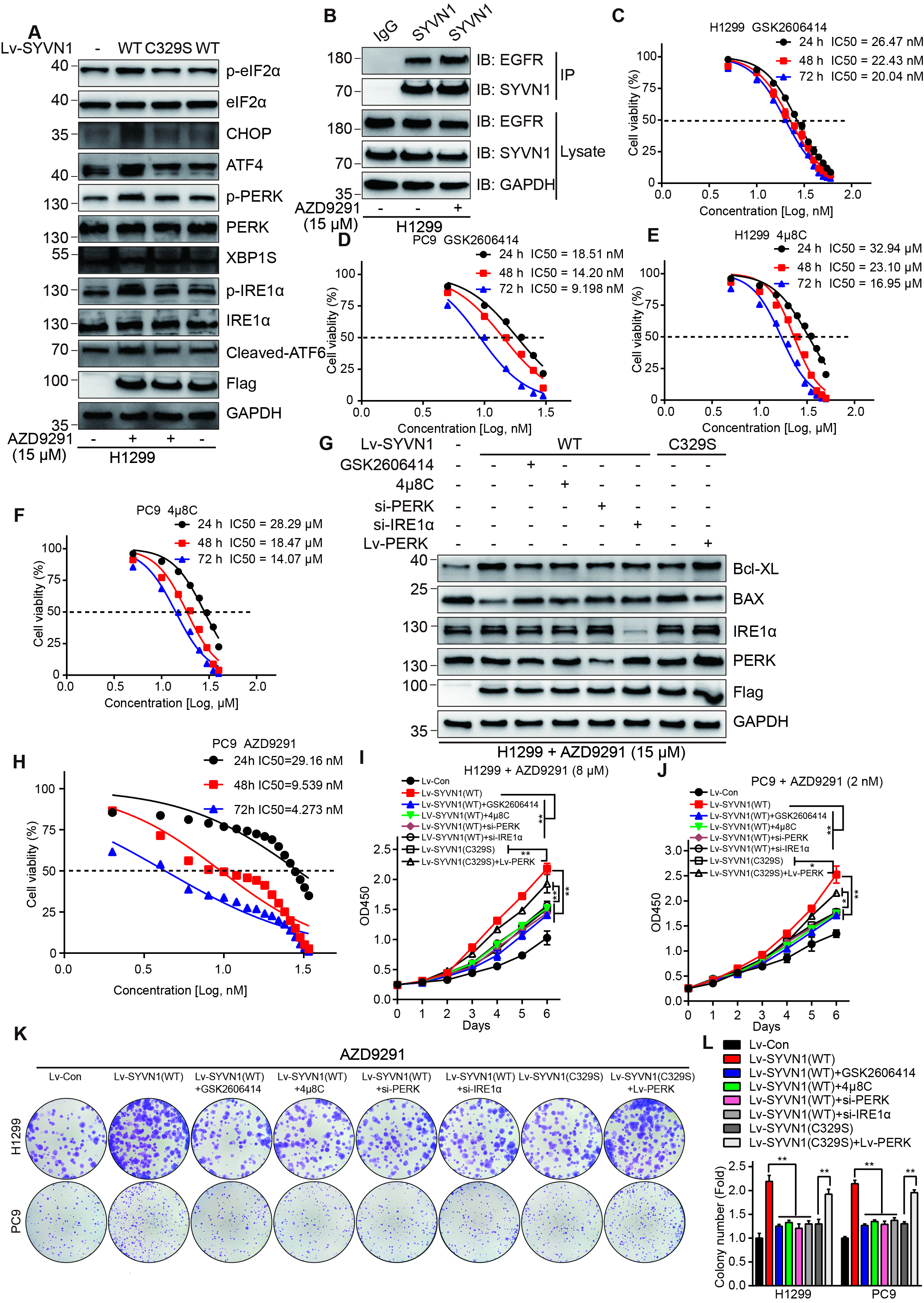

Supplement: Supplementary file 6 — Supplementary Figure S5 [file 41419_2025_7978_MOESM6_ESM.jpg]

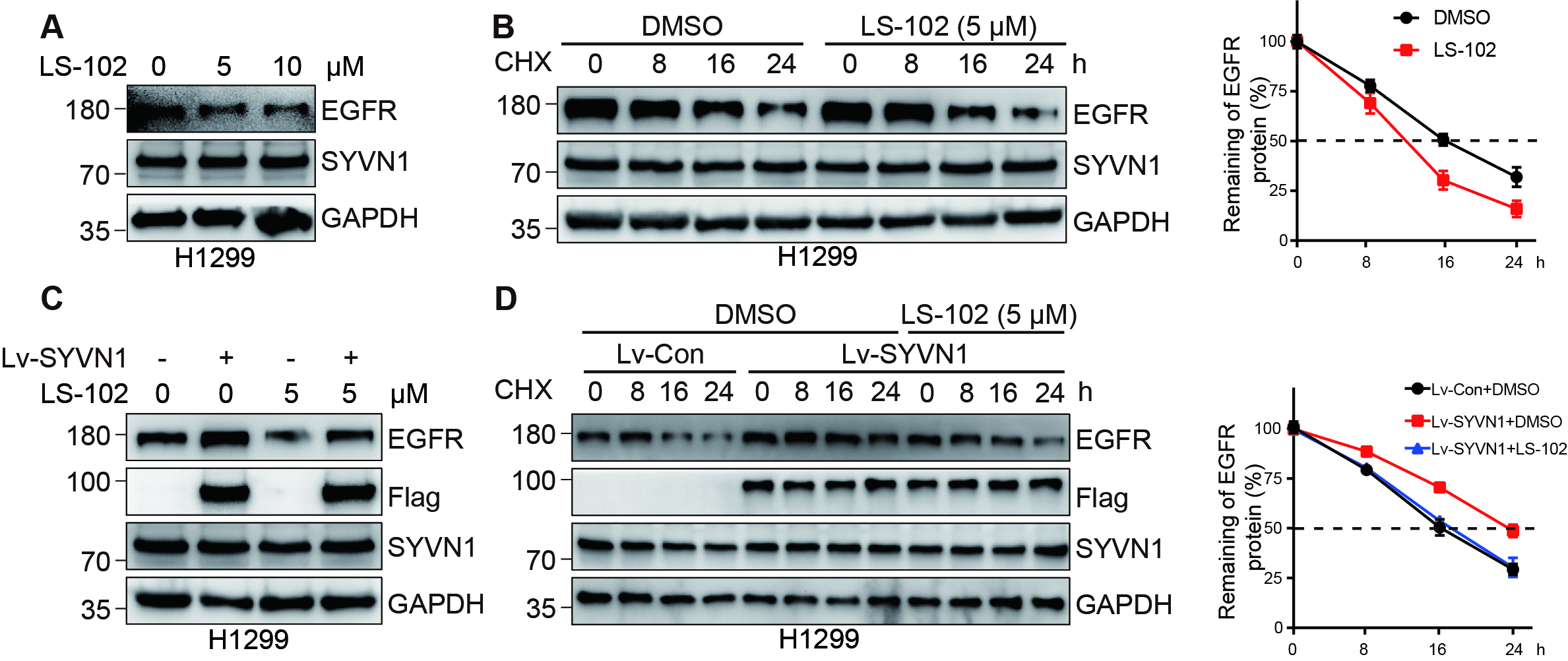

Supplement: Supplementary file 7 — Supplementary Figure S6 [file 41419_2025_7978_MOESM7_ESM.jpg]

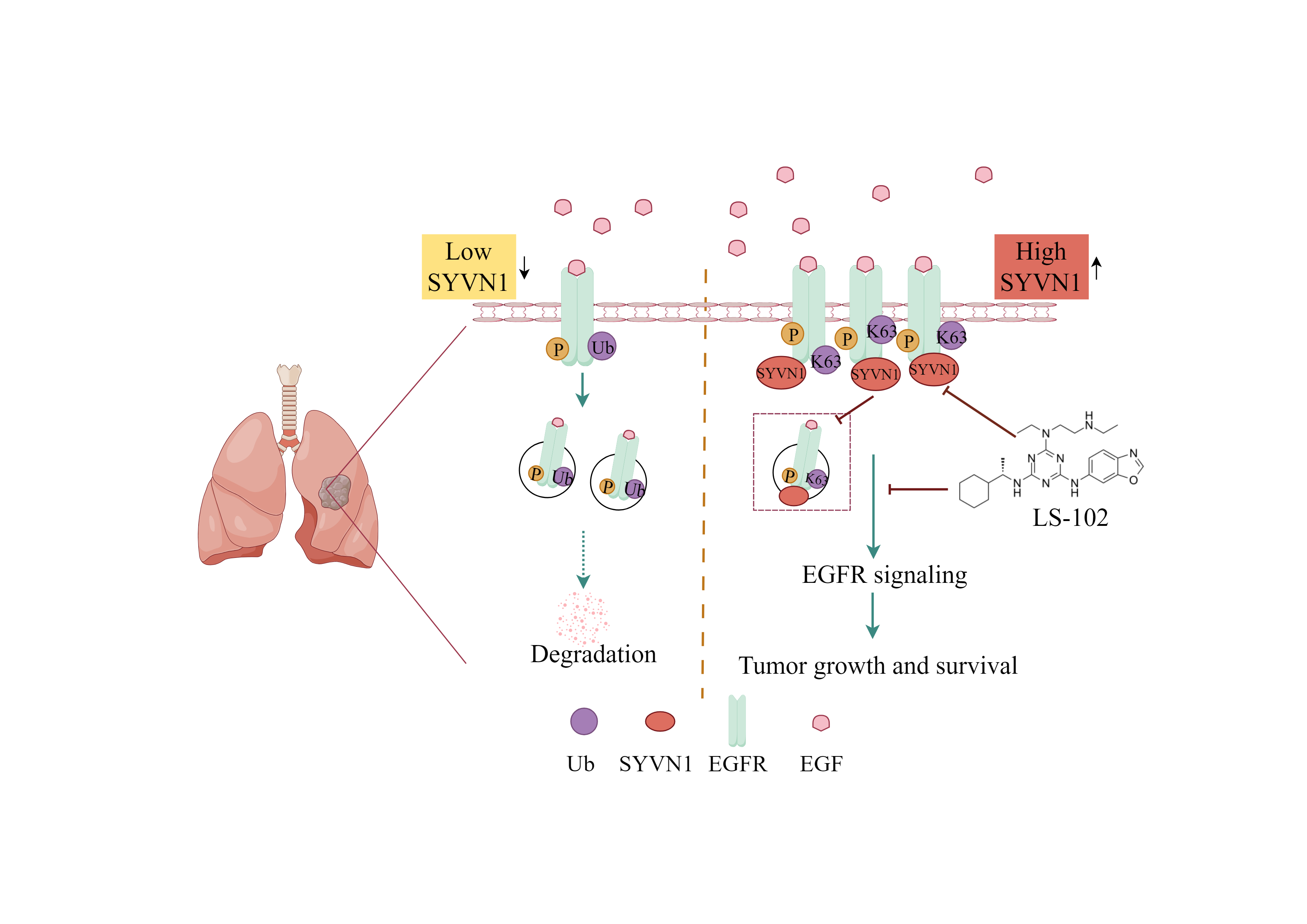

Supplement: Supplementary file 8 — Supplementary Figure S7 [file 41419_2025_7978_MOESM8_ESM.png]
