# Supplementary material for: Targeting the SYVN1-EGFR axis: a breakthrough strategy for TKI-resistant NSCLC
Source: Cell Death Dis. 2025 Aug 28;16(1):655. doi: 10.1038/s41419-025-07978-2 (PMC12394631; doi:10.1038/s41419-025-07978-2)
Supplement: Supplementary file 9 — Table S1–3 [file 41419_2025_7978_MOESM9_ESM.docx]

**Supplementary Table S1. Differential expression of SYVN1 in NSCLC and adjacent tissues**

|  | n | SYVN1 expression | | Chi-square  Value | p value |
| --- | --- | --- | --- | --- | --- |
|  |  | High(%) | Low(%) |  |  |
| NSCLC | 90 | 38 | 52 | 48.169 | 0.0000 |
| Adjacent tissues | 90 | 0 | 90 |  |  |

**Supplementary Table S2. Correlation between SYVN1 expression and clinicopathological characteristics**

|  | variables | SYVN1 expression | | total | χ2 | p value |
| --- | --- | --- | --- | --- | --- | --- |
|  |  | Low | High |  |  |  |
| Age (year) |  |  |  |  | 0.228 | 0.633 |
|  | <60 | 22 (24.4%) | 18  (20%) | 40 |  |  |
|  | ≥60 | 30 (33.4%) | 20 (22.2%) | 50 |  |  |
| Sex |  |  |  |  | 0.294 | 0.588 |
|  | Female | 23 (25.6%) | 19 (21.1%) | 42 |  |  |
|  | male | 29 (32.2%) | 19 (21.1%) | 48 |  |  |
| Histologic grade |  |  |  |  | 1.966 | 0.161 |
|  | Ι/II | 34 (37.8%) | 30 (33.3%) | 64 |  |  |
|  | III | 18 (20%) | 8 (8.9%) | 26 |  |  |
| T stage |  |  |  |  | 2.277 | 0.131 |
|  | T1/T2 | 44 (48.9%) | 36  (40%) | 80 |  |  |
|  | T3/T4 | 8 (8.9%) | 2 (2.2%) | 10 |  |  |
| N stage |  |  |  |  | 2.182 | 0.140 |
|  | N0 | 29 (32.2%) | 27  (30%) | 56 |  |  |
|  | N1/N2/N3 | 23 (25.6%) | 11 (12.2%) | 34 |  |  |
| TNM stage |  |  |  |  | 1.575 | 0.210 |
|  | Ι/II | 38 (42.2%) | 32 (35.6%) | 70 |  |  |
|  | III | 14 (15.5%) | 6 (6.7%) | 20 |  |  |
| Primary tumor size |  |  |  |  | 1.943 | 0.163 |
|  | <3 cm | 28 (31.1%) | 26 (28.9%) | 54 |  |  |
|  | ≥3 cm | 24 (26.7%) | 12 (13.3%) | 36 |  |  |
| Anatomic neoplasm subdivision |  |  |  |  | 0.167 | 0.682 |
|  | Left | 17 (18.9%) | 14 (15.5%) | 31 |  |  |
|  | Right | 35 (38.9%) | 24 (26.7%) | 59 |  |  |
| OS event |  |  |  |  | 4.942 | 0.012 |
|  | Alive | 21 (23.4%) | 7  (7.8%) | 28 |  |  |
|  | Dead | 31 (34.4%) | 31 (34.4%) | 62 |  |  |

* Statistically significant(*p*<0.05)

**Supplementary Table S3. Univariate and multivariate analyses of the factors correlated with Overall survival of NSCLC patients**

| variables | Univariate analysis | | |  | Multivariate analysis | | |
| --- | --- | --- | --- | --- | --- | --- | --- |
|  | HR | 95%CI | p value |  | HR | 95%CI | p value |
| SYVN1 expression (High vs. Low) | 2.05 | 1.05-4.00 | 0.035 |  | 1.91 | 0.98-3.71 | 0.058 |
| Sex (Male vs. Female) | 1.40 | 0.75-2.59 | 0.295 |  |  |  |  |
| Age (≥60 vs. ＜60) | 1.12 | 0.62-2.02 | 0.708 |  |  |  |  |
| Histologic grade (III vs. Ι/II) | 1.50 | 0.85-2.65 | 0.164 |  |  |  |  |
| T stage (T3/T4 vs. T1/T2) | 1.68 | 0.84-3.34 |  |  |  |  |  |
| N stage (N1-3 vs. N0) | 1.55 | 0.86-2.78 | 0.142 |  |  |  |  |
| TNM stage (III vs. Ι/II) | 2.12 | 1.11-4.05 | 0.023 |  | 2.5 | 1.28-4.90 | 0.008 |
| Primary tumor size (≥3 vs. ＜3) | 1.36 | 0.76-2.44 | 0.300 |  |  |  |  |
| Anatomic neoplasm subdivision (Right vs. Left) | 0.93 | 0.53-1.64 | 0.806 |  |  |  |  |

* Statistically significant(*p*<0.05)
